# Fine-grained information extraction from German transthoracic echocardiography reports

**DOI:** 10.1186/s12911-015-0215-x

**Published:** 2015-11-12

**Authors:** Martin Toepfer, Hamo Corovic, Georg Fette, Peter Klügl, Stefan Störk, Frank Puppe

**Affiliations:** 1 Chair of Computer Science VI, University of Würzburg, Am Hubland, Würzburg, D-97074 Germany; 2 Comprehensive Heart Failure Center, University of Würzburg, Straubmühlweg 2a, Würzburg, D-97078 Germany; 3 Averbis GmbH, Tennenbacher Straße 11, Freiburg, D-79106 Germany

## Abstract

**Background:**

Information extraction techniques that get structured representations out of unstructured data make a large amount of clinically relevant information about patients accessible for semantic applications. These methods typically rely on standardized terminologies that guide this process. Many languages and clinical domains, however, lack appropriate resources and tools, as well as evaluations of their applications, especially if detailed conceptualizations of the domain are required. For instance, German transthoracic echocardiography reports have not been targeted sufficiently before, despite of their importance for clinical trials. This work therefore aimed at development and evaluation of an information extraction component with a fine-grained terminology that enables to recognize almost all relevant information stated in German transthoracic echocardiography reports at the University Hospital of Würzburg.

**Methods:**

A domain expert validated and iteratively refined an automatically inferred base terminology. The terminology was used by an ontology-driven information extraction system that outputs attribute value pairs. The final component has been mapped to the central elements of a standardized terminology, and it has been evaluated according to documents with different layouts.

**Results:**

The final system achieved state-of-the-art precision (micro average.996) and recall (micro average.961) on 100 test documents that represent more than 90 % of all reports. In particular, principal aspects as defined in a standardized external terminology were recognized with *f*_1_=.989 (micro average) and *f*_1_=.963 (macro average). As a result of keyword matching and restraint concept extraction, the system obtained high precision also on unstructured or exceptionally short documents, and documents with uncommon layout.

**Conclusions:**

The developed terminology and the proposed information extraction system allow to extract fine-grained information from German semi-structured transthoracic echocardiography reports with very high precision and high recall on the majority of documents at the University Hospital of Würzburg. Extracted results populate a clinical data warehouse which supports clinical research.

**Electronic supplementary material:**

The online version of this article (doi:10.1186/s12911-015-0215-x) contains supplementary material, which is available to authorized users.

## Background

Information extraction in the clinical domain aims to translate textual reports into structured representations. It enables semantic information retrieval, the application of formal knowledge to patient management, and further data analysis like clinical research based on statistics and evidence based medicine. While some data for patient management already exists in a coded format, e.g., lab data or the ICD [1] codes of diagnoses, the majority of patient information is still only available as textual documents like discharge letters or reports from specific examinations like echocardiography or radiology. The main purpose of these documents is communication among different physicians, but they are also a valuable source of detailed patient information. Therefore, information extraction from clinical documents has received much attention [2–6].

Since clinical reports often have a telegram-style consisting of noun phrases with many technical terms that have semantic constraints, ontology-driven information extraction methods are promising [2, 7], that is, systems that make active use of ontologies (terminologies) [8]. Standardization efforts like the above mentioned ICD, SNOMED [9], UMLS [10], LOINC [11], or MeSH [12] are valuable sources of knowledge for biomedical text processing in general, yet, custom terminologies or at least extensions to existing vocabularies are necessary under certain circumstances. First, availability and coverage of non-English languages lack behind their English counterparts for most standardization efforts, for instance, in German [13]. Several researchers identified the gap between observed terms and shared terminologies as well as missing properties of lexical entries as major problems for applications, e.g., to identify pathological findings in German radiology reports [14]. Second, more fine-grained models than provided by shared general conceptualizations may be required for special report types, even in English. For instance, Friedlin [15] found that representations produced by the UMLS MetaMap [16] program were not adequate, tested on chest x-ray reports, discharge summaries, and admission notes. The main sources of error were: different conceptual specificity, missing synonyms, and missing conceptual representation. The application of custom domain-specific dictionaries and thesauri avoids such deficiencies, however, their development is costly. As a result, many clinical domains and languages lack appropriate representations and tools. Clinical terminology extraction and ontology learning are active areas of research, especially for non-English research groups like, for example, Marciniak et al. [17], to overcome this problem.

In this work, we address information extraction from German transthoracic echocardiography (TTE) reports with a broad coverage of relevant concepts. We constructed a specialized terminology (see Additional file 1) which has been developed in a data-centric way on documents of the University Hospital of Würzburg. In order to support a standardized user-friendly view on the data, we manually mapped entries to an experts’ conceptualization of the domain instead of applying a more general terminology. In this work, we used the guideline provided by Voelker et al. [18], which is a recommendation of the German Cardiac Society for the structure of echocardiography reports in German. Although the guideline can be used to structure and rank concepts according to their relevance, the publication does not contain synonyms, hence, it is not suitable for direct application in an information extraction system.

**Fig. 1:**
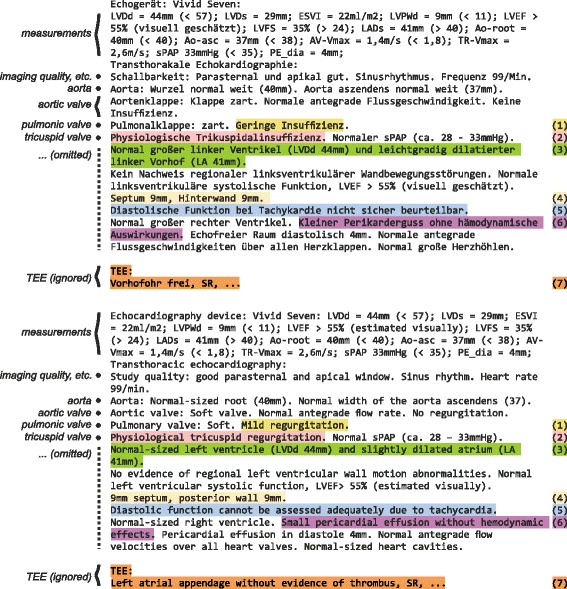
Example of a semi-structured echocardiography report (top: German, bottom: English translation). Visual appearance and composition is determined by a layout, for instance, this document has a list of numeric parameters at the top, followed by different subsections (see descriptions on the left side). Selected challenging sections are emphasized and enumerated on the right hand side. (1): Attribute ambiguity. (2): Object-attribute compound. No subsection header. (3): Enumeration. (4): Enumeration. (5): Nested prepositional phrase. (6): Negation. (7): Out ouf domain subsection
- “The patient reports dyspnea when exercising. He suffers from frequent cough with sputum. He has no edema in arms, legs and body.”

The system used for information extraction in this work is composed of modules designed for reusability and operates on semi-structured clinical documents like the report shown in Fig. 1, written with constituents as in Example 1 a) instead of grammatically correct sentences like Example 1 b).

- Exertional dyspnea, frequent cough with sputum, no edema in arms, legs and body.

The algorithm is able to immediately integrate new concepts into the extraction component and to apply it from scratch without retraining a model or annotating training examples. This contrasts to supervised machine learning approaches for clinical information extraction.

**Fig. 2:**
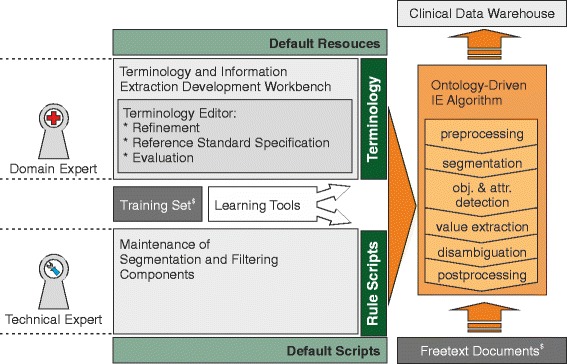
Overview of the terminology development and information extraction setting. Based on default resources (dictionaries, templates, etc.) and supported by automatically inferred concept proposals, domain experts iteratively refine the domain knowledge and the high-level extraction knowledge of the terminology for each clinical subdomain (top left); technical experts adapt preexisting segmentation and filtering rules to the needs of specific subdomains (bottom left). The terminology and the segmentation module are integrated into a generic ontology-driven information extraction method that keeps the same across domains (mid right). It populates extracted attribute value pairs into a clinical data warehouse (top). $: input documents are (pre)processed by a de-identification module in order to ensure patient privacy

**Fig. 3:**
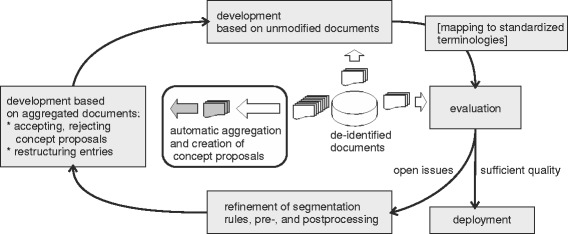
Process model. Most entries of the terminology originate from a large amount of de-identified documents that are automatically aggregated into more compact files which are the basis for automatically created concept proposals. Development on de-identified documents that were not aggregated allows to further refine the terminology and to detect quality issues. If required, concepts are mapped to standardized external resources. If subsequent evaluation reveals open issues, refinement of segmentation components or other computational aspects can be requested and a new development iteration starts. When all components perform sufficiently, the final information extraction component is deployed and populates a clinical data warehouse

As outlined in Fig. 2, the central elements of the intended terminology development and information extraction setting are the terminology of the clinical subdomain, a domain expert, a technical expert, a collection of clinical documents (training set), and algorithmic components for terminology learning (learning tools), refinement support (terminology editor), segmentation (rule scripts), and a generic ontology-driven information extraction algorithm. Mappings to external conceptualizations can be used to create standardized views on the data as depicted in Fig. 3. Documents are de-identified in order to preserve patient privacy. Finally, deployed information extraction modules can be used to populate a clinical data warehouse either directly in a clinical data warehouse environment like [19] or integrated as a text mining service into a cloud infrastructure like [20].

The following paragraphs offer an overview of related work on clinical information extraction. Section ‘Methods’ describes our approach and the tool support. Section ‘Results and discussion’ presents experimental results. Finally, Section ‘Conclusions’ gives a summary and an outlook.

### Related work

First, common clinical text processing architectures are described, and different comprehensive clinical information extraction systems are compared. These systems typically reuse existing natural language processing libraries from other projects in combination with special components to serve a lot of different tasks. Second, this section reviews clinical information extraction approaches that were tailored to specific subdomains, and approaches that can be tuned to process documents with shallow structure. Third, we sum up previous research on German-language clinical natural language processing and information extraction. Table 1 provides an overview of results of different systems.

**Table 1:** Overview of selected clinical information extraction system evaluations; see Section ‘Related work’

Article	Year	Domain	Language	Test set	Concepts	Prec.	Rec.	F1
[28]	2005	Echo	English	408 doc.	10	.99	.78	.87
[29]	2012	Echo	English	475 doc.	4 ^*a*^	.95	.89	.92
[7]	2009	Mammography	Polish	705 doc.	66	.996	.995	.996
[7]	2009	Diabetes	Polish	100 doc.	68	.993	.965	.979
[5]	2010	General	English	160 doc.	many ^*c*^	.801 ^*b*^	.645 ^*b*^	.715 ^*b*^
[24]	2004	General	English	150 sent.	many ^*c*^	.89	.77	- ^*d*^
[40]	2009	Metastatic Tumor	English	101 doc.	many ^*c*^	.73	.58	.65
[40]	2009	Primary Tumor	English	101 doc.	many ^*c*^	.80	.84	.82
[40]	2009	Anatomical Site	English	101 doc.	many ^*c*^	.97	.98	.97
[14]	2013	Radiology	German	40 doc.	2 ^*e*^	.54	.74	.63

Year: year of publication. Domain: intended domain or the domain used for evaluation. Test Set: size of test set used for evaluation, i.e., number of documents/sentences. Concepts: number of classes, concepts or terminology used for reported results. ^*a*^concept level analysis, see related work for details. ^*b*^named entity recognition results used as an upper estimate; see original work for more detailed figures. ^*c*^application uses standardized resources such as UMLS or ICD-O with a large number of concepts. ^*d*^omitted to reflect that precision and recall have been evaluated on different sets of sentences. ^*e*^Sentence-level classification of normal vs. pathological findings

In the past decades, several general systems for medical and in particular clinical information extraction have been introduced: MedLEE [3], MEDSYNDIKATE [4], HITEx (Health Information Text Extraction) [6], SeReMed [2], or Apache cTAKES (Clinical Text Analysis and Knowledge Extraction System) [5] – just to name a few. Most of them follow a canonical design of document processing stages. They first segment the document into units like sections, sentences, add part-of-speech tags, and split sentences into chunks, especially noun phrases. Dictionary-based annotators like ConceptMapper [21] are applied to find clinical concepts using manually curated lexical expressions that refer to the concepts, and map them to unique identifiers. Search may be limited to match terms only inside the same noun phrase. Typically, pipelines contain further processors to detect if concepts are negated, time dependent, or refer to family history, for instance, using regular expressions [22]. Separate extractors may be integrated for specially structured information like medication [23]. The final pipeline components perform post-processing operations like information aggregation.

In the work of Friedman et al. [24], the output of a medical NLP system (MedLEE) was utilized to automatically map clinical documents to UMLS codes. The application achieved.89 precision on 150 randomly selected sentences, and it obtained a recall of.77 with respect to UMLS coding of all terms. The authors note that UMLS modifiers lacked granularity and coverage with respect to clinical purposes, especially regarding degree, change, and temporal information.

HITEx and Apache cTAKES both use open-source libraries like WEKA [25] or MALLET [26] to perform some tasks based on machine learning methods. Nevertheless, regular expressions and rule-based components still play a central role in both systems. The same applies to the approach of Mykowiecka et al. [7] who make use of a general rule-based information extraction system to create components for Polish mammography reports and hospital records of diabetic patients. They use typed feature structures that are combined by manually written grammar rules to fill in templates defined in a domain ontology. Pre-processing includes common tasks like tokenization, morphological analysis, and lexicon lookup. Post-processing addresses word sense disambiguation, combining isolated single extractions into more complex structures based on syntactic segments and ontology types, as well as coordination and anaphoric expression handling. The main grammar rules are responsible for negation detection, certain kinds of coordination, and to resolve some aspects of word sense disambiguation. As depicted in rows three and four of Table 1, the results reported for their system show that rule-based information extraction performs well on clinical subdomains. The downside of the approach is the demand for substantial rule engineering.

Information extraction approaches for specific clinical subdomains have been in the focus of research for several years, e.g., to extract smoking status [27]. There have also been several studies that investigated information extraction from English echocardiography reports.

For instance, published in 2005, Chung and Murphy [28] extracted concept-value pairs and evaluated their system on ten clinical concepts: aortic valve stenosis, cardiac shunt, ejection fraction, intracardiac thrombus, left ventricular hypertrophy, mitral valve insufficiency, mitral valve prolapse, pericardial effusion, pulmonary hypertension, and valvular vegetations (cf. 1st row of Table 1 for results). Their approach uses manually defined extraction patterns that operate on the output of a concept mapper using a standardized medical terminology. The work of Garvin et al. [29] from 2012 focused on extracting one specific type of information (ejection fraction) at the document level which relates to 4 concepts at the class level. They studied a collection of documents from different medical centers with different degrees of structure: unstructured, semi-structured, and structured reports. They used regular expressions and rules which produced sufficient performance at the concept level (cf. 2nd row of Table 1) to accurately recognize the class at the document-level (99.2 % F1). In contrast to these systems, the application evaluated in this paper aims at wide-coverage information extraction. It operates on more than 440 attribute value pairs (more than 150 attributes) in total for the echocardiography domain. Furthermore, it provides a resource for reports in German.

Most research on clinical natural language processing and information extraction addressed English-language documents. German-language applications have to cope with limited supply of tools and libraries, and there are less resources like terminologies or annotated corpora. Schulz et al. [13] published a study about German-language content in biomedical resources in 2013. They found that several resources were available but that their extend was typically behind their English counterparts. The most comprehensive resource was the German SNOMED CT translation which had far more entries than other resources. However, they state that it was “outdated and not officially available”.

There has also been research considering clinical natural language processing tasks in German, for instance, sentence boundary and abbreviation detection [30] or part-of-speech tagging [31]. In 2002, Hahn et al. [4] described a system for the extraction of information from findings reports, called MEDSYNDIKATE, which heavily builds upon syntactic parsing and handcrafted or automatically assembled domain knowledge. Evaluation was performed by analysis of three syntactic settings (genitives, prepositional phrases, modal verbs or auxiliaries) with encouraging results. There is considerable overlap between the ideas behind their system and the application used in this work. However, in order to cope with limited initially available domain knowledge and grammar resources, we chose a system design that is restricted to surface syntactic properties and a light-weight knowledge representation.

An approach that is also similar in mind was conducted by Denecke [2] in 2008. It uses the UMLS to structure and extract information from medical documents by transforming shallow syntactic structure to semantic structure with rules. Evaluated on English cancer-related chest x-ray reports, the system extracted findings with 93 % precision and 83 % recall. The system has also been applied to German clinical narratives; it achieved 93 % precision and 92.5 % recall in a preliminary evaluation [32] concerning hospitalization and admission diagnosis information in 20 surgical letters. In 2013, Bretschneider et al. [14] used a sublanguage grammar for a binary classification task on German pathology reports of lymphoma patients. They aimed to filter out only the relevant pathological findings and to disregard normal observations at a sentence level. Sentences containing both types of findings were regarded as being completely pathological. Their approach builds upon a standardized terminology (RedLex – German Version [33]) but the vocabulary had to be extended by a corpus-based learning step. They finally reached 74.3 % recall which significantly surpassed the baseline’s recall (3.7 %). Precision was 54.4 % while the chosen baseline reached 100 %. Insufficiency of the applied vocabulary was identified as a still present major issue to be addressed in the future.

A recent work on processing German patient records has been contributed by Krieger et al. [34], in which they sketch an interesting approach, centered on natural language processing aspects. Two small experiments have been performed for parsing and relation extraction. To the best of our knowledge, detailed information extraction results of this approach have not been published yet.

Previous work [19] reported on a machine-learning approach for information extraction from clinical documents with Conditional Random Fields (CRF) [35]. Although the precision was very good, additional methods are required. The most fundamental problem hindering practical use of such an application of CRFs in our setting stems from their supervised machine learning nature. Appropriate methods for interactive information extraction and terminology development like intended in this paper must not depend on large amounts of manually created annotations or a fixed conceptualization of the domain because both – terminology and annotations – may change frequently during development. Supervised machine learning, however, requires a stable specification of the classes, that is, domain concepts, and a sufficient amount of annotated training data. As a result, both requirements are not satisfied. Moreover, one has to carefully design the encoding of states in the model in order to avoid efficiency problems and to achieve good generalization performance. This task is not trivial and may especially vary across subdomains for optimal results. Encodings that mirror fine-grained concept identifiers [19] have inherent performance issues. However, coarse encodings have been used successfully for clinical named entity recognition, for example, in Swedish [36], which is a promising approach to support terminology development as well as semantic interpretation.

## Methods

### Project overview

Figure 1 shows an artificial echocardiography report which is representative for the types of reports addressed in this paper. It will be referred to in the following paragraphs. According to Garvin et al.’s [29] notion of degree of document structure, the shown example is semi-structured because it has a moderate degree of alignment and organization. By contrast, structured reports have tabular form, while reports written in free text are called unstructured. In general, measurements and some interpretations of reports at the University Hospital of Würzburg are generated directly from a machine while physicians add further interpretations and are free to edit the generated part of the text.

Figure 2 depicts how we built the application that extracts information in this setting. It has two central aspects. On the one hand, *terminology construction* has been performed by a domain expert and technical staff with special tool support (terminology editor). The former iteratively specified the relevant concepts with basic “ontology learning & refinement” assistance. The latter adapted segmentation rules and provided technical training and support. The other core component is the generic *ontology-driven information extraction algorithm* for semi-structured domains that is essentially controlled via terminology structure and the concepts’ properties.

Finally, terminology reordering and mapping on a reference guideline for German echocardiography reports was performed.

### Terminology model

The key task for the domain expert is to build a structured terminology consisting of objects, attributes and values with appropriate usage of generalized classes (templates), dictionaries, and variants. Table 2 lists the main concept types used in the terminology along with examples.

**Table 2:** Knowledge representation: main types of concepts

Concept type	Description	Examples
*Structure*	organization of entries; no meaning for information extraction	measurements, assessment
*Object*	anchor for ambiguous attributes; definition of a complex frame	aortic valve, mitral valve
*Object attribute*	type of information with ambiguous meaning; it requires an object context	regurgitation (aortic)
*Attribute*	unambiguous type of information stated in reports	LVEF, E/E’
*Value*	specific state of an attribute	present, absent, severe

The most fundamental kinds of entries are *variants* (see Table 3). In form of either a string or a regular expression they specify lexical expressions that refer to *concept* entries, which represent the semantic units that are stated in reports. In order to keep things simple for domain experts, there are only three main types of concepts that were used in this work: objects, attributes and values. Concepts with a rich internal structure and many properties are modelled as *objects*. They accept attributes which have certain kinds of *values*. While some *attributes* like measurements are typically unambiguous and can be recognized without context, *object attributes* require a resolving object context. In particular for constructing a terminology in German, variants can be defined as being attribute-value (av) or object-attribute (oa) compounds. Consider, for example, the object-attribute-value constellation: “mitral valve”, “mitral valve regurgitation”, “severe mitral valve regurgitation”. Since “regurgitation” is ambiguous and may refer to different kinds of objects, it must be entered into the terminology as an object attribute with a standard variant “Insuffizienz” (engl.: regurgitation). However, in German there are also expressions that directly point to a specific kind of regurgitation, for example, the object-attribute compound “Mitralklappeninsuffizienz” of the object attribute that represents regurgitation.

**Table 3:** Knowledge representation: the main types of variants are “standard” (std) and “regular expression” (regexp)

Variant type	Description	Example (GER)	Example (ENG)	Compound
*std*	simple string	Aortenklappe	aortic valve	
*std*	simple string	Insuffizienz	regurgitation	
*std*	simple string	Aortenklappeninsuffizienz	aortic valve regurgitation	oa
*regexp*	regular expression	|(= s*)?[0-9]+|	|(= s*)?[0-9]+|	

Each entry can optionally be specified as an object-attribute (oa) compound, or an attribute-value (av) compound

Finally, there are certain aspects of the terminology model that allow for better and more convenient organization and management of entries. In order to increase human readability in large terminologies, concepts can be grouped by *structure* nodes, and variants can be centrally stored in *dictionaries*. Similarly, redundancy in value definitions can be avoided by the use of *templates* which allow for sharing definitions of attributes with similar semantics. Template reference can be seen as a kind of semantic class membership or a light-weight “is-a” relation. For instance, the attribute “mitral valve regurgitation” references the template “regurgitation” which states that it is a special kind of regurgitation, and that this attribute should accept the same values as specified for the more general attribute.

Despite of these main types and their properties, there are additional aspects that can be specified in the terminology to control the information extraction algorithm.

### Initial terminology development

**Fig. 4:**
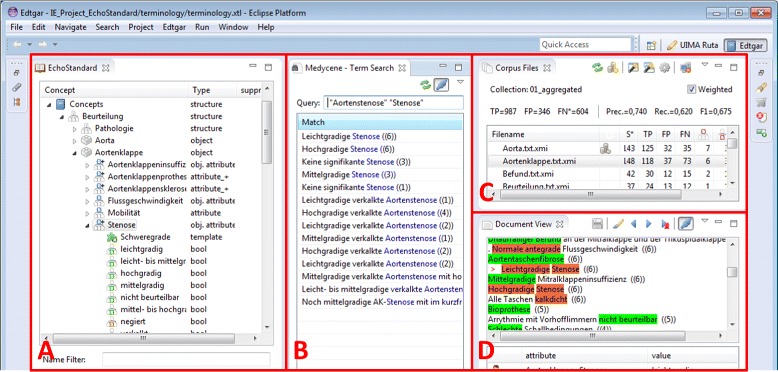
Integrated terminology development and information extraction workbench. **a** terminology editor, **b** query and search tool for free strings, terminology concepts and annotations, **c** document collection view, **d** annotation editor for documents from the collection in (**c**)

Terminology acquisition was assisted by a tool to be used by domain experts for integrated terminology construction, terminology management, information extraction, reference standard (gold standard) annotation and evaluation (Fig. 4). A predecessor of the system has been described in [37]. The terminology as shown to the user is depicted in Fig. 4a. The software is especially tailored to support the domain expert’s process model that is shown in Fig. 3. It consists of a few general steps: initial automatic aggregation of training documents and generation of concept proposals, terminology refinement based on aggregated documents, terminology refinement based on unmodified documents, mapping of concepts to standardized terminologies (optional), evaluation, optional: request for improvement of segmentation or pre-/postprocessing rules and start of a new refinement iteration. When the system reaches sufficient quality, it is deployed and integrated into the clinical data warehouse system.

Based on previous work with clinical documents, we observed that many types of reports contain highly redundant phrases, i.e., expressions that have a high frequency conditioned on the domain and the specific hospital. This may be caused by the nature of the reports. For example, they often contain examinations which follow local guidelines with mandatory statements about physical conditions, and each clinician has its own but typically consistent preferences to create a report; sometimes assisted by custom templates of office applications. As a consequence, the first document-centric interaction of the user with the system is based on so-called *aggregated documents* before it moves to the original reports. Aggregated documents contain all distinct phrases that occur in the whole training corpus along with their frequencies. Tokens are normalized, for instance, numbers are replaced by the string “9”. In order to preserve contextual information that may be required for disambiguation, segments are grouped per subsection. As an example, if the ambiguous phrase “Severe stenosis” appeared 6 times in subsections of the type “aortic valve” in the corpus, the aggregation file for “aortic valve” contexts will contain a line “Severe stenosis ((6))” as shown in Fig. 4d (German: “Hochgradige Stenose (6)”). Aggregation reduces the number of different phrases a domain expert has to inspect and reveales the importance of covering a phrase in the terminology through frequency information. The view marked “C” in Fig. 4 shows the list of all aggregated files for the different contexts, where the file “Aortenklappe.txt.xmi” (aortic valve) is selected for further processing. A part of this file is shown in Fig. 4d.

In each refinement iteration, the current terminology is used for automatic annotation. From each segment, we extracted attribute candidates (nouns or noun groups filtered by various word lists) which are presented to the user, who accepts or rejects them (this step is not exemplified in Fig. 4). For each accepted attribute, all phrases containing this attribute are displayed to the domain expert in Fig. 4b (all variants for the attribute “Aortenstenose” (aortic valve stenosis)) to decide about the different values of the attribute and their synonyms and semantic properties (e.g. regular expressions) in the terminology. For recurrent values of different attributes like negation or degree of severity, we provide templates covering the typical variety of these values so that the user can assign a template to an attribute.

Internal feedback on the quality of the current state of the system regarding training instances is required to know if one can proceed to evaluation on a test set. For this purpose, verified annotations are created on the training set and compared to automatic output. The domain expert can either discard segments of a document as irrelevant (i.e. containing no relevant information), mark segmentation errors, or create reference standard annotations manually or semi-automatically. For instance, the domain expert checks all automatically extracted information as either correct or incorrect with an editor (see Fig. 4d) and adds missing attribute-value pairs (segments contain often more than one annotation). The latter is the most time consuming step. For each error, the terminology is appropriately modified (in Fig. 4a).

If the terminology is sufficiently developed with regard to aggregated training documents, it is evaluated against a new collection of unmodified documents. In this step, the most time-consuming task is the definition of a reference standard in the new document collection. To speed up this step, human annotators can choose a semi-automatic process that just requires validation and modification of proposed attribute value pairs. In Fig. 4c, the numbers show the results of comparing extractions made by the system to reference standard annotations (FP, TP. FN; here for the aggregated files, in the real evaluation the original unmodified files are used instead of the aggregated files).

If necessary, the terminology and also the generic segmentation rules can be improved and a new evaluation with new documents must be performed.

Since the information extraction component is implemented with a generic algorithm that directly infers the connections between text and the available terminology, no training process or similar time-consuming computations have to be performed as it would be the case for supervised machine learning approaches.

### Segmentation and ontology-driven information extraction

The information extraction pipeline implemented in the tool is based on Apache UIMA [38]. The core extraction logic is implemented in Java. It uses a deterministic search over the document structure to perform a disambiguation of terms with multiple meanings. It relies on properties of concepts, variants, and relations defined in the terminology. Pre- and postprocessing operations are carried out through customizable Apache UIMA Ruta [39] rule scripts.

The central information extraction logic can be summarized by different stages as outlined on the right hand side of Fig. 2: preprocessing, segmentation, object and attribute extraction, value extraction, disambiguation with contexts, postprocessing with filters and mappings.

First, the system compiles the terminology into special data structures for efficient candidate detection and retrieval of all possible word senses of candidate terms. For example, a rule script for regular expressions and a trie-based word list are created that together cover all variants of all concepts of the terminology. The next stage consists of general and domain-specific document segmentation scripts. These scripts have a default initialization but they are configurable and adaptable to satisfy special needs on certain subdomains. The most important aspects of the output of this stage are subsections, representing contexts of object concepts, and segment annotations within each subsection. Since errors of this component are propagated to subsequent processors, it constitutes a crucial part of the pipeline. In Fig. 1, Example 1, it is necessary to recognize the subsection relating to the pulmonary valve correctly, otherwise the ambiguous phrase *“Geringe Insuffizienz”* may be interpreted as part of the aortic valve subsection. In some cases, subsections are not separated clearly by subsection headers as can be seen in Fig. 1, Example 2, where object-attribute compounds can provide necessary contexts for disambiguation. After segmentation, the generic concept extraction and assignment component iterates over subsections and segments, and recognizes objects and attributes within the segments, and assigns values taking into account contexts for disambiguation of terms with multiple meanings. Finally, post-processing operations with filters for reducing redundancy and mappings for aggregating terms further refine the output. The following paragraphs describe certain aspects of the algorithm in more detail.

As noted before, the majority of segments in the echocardiography reports showed very simple syntactic composition; most of them can be recognized with regular expressions. Some phenomena, however, need to be addressed even in semi-structured domains, for example, because certain symbols like commata are used to separate segments and also occur in enumerations. For instance, the text passage *“Septum 9mm, Hinterwand 9mm.”* in Fig. 1 (Example 4) contains two different segments separated by comma, while *“Unauffälliger Befund an A, B und C”* has to be conjoined. The segmentation rules cover several cases of enumeration where segments need to be merged. By contrast, sentences like *“Normal großer linker Ventrikel (LVDd 44mm) und leichtgradig dilatierter linker Vorhof (LA 31mm).”* (Fig. 1, Example 3) need to be split into two segments with special segmentation rules in order to separate their statements (Fig. 1 offers translations for all examples in German written in italic with the exception of “unauffälliger Befund bei A, B und C” (no findings at A, B, and C)).

There are some formulations that require at least a shallow parse that creates chunks to be handled correctly, for instance, the simple negation phrase in Example 6, Fig. 1. Furthermore, consider the nested prepositional phrase in *“Diastolische Funktion [bei Tachykardie] nicht sicher beurteilbar”* (Fig. 1, Example 5). In this example, tachycardia is present, but the diastolic function cannot be assessed adequately. Simple key phrase matching assigns “cannot be assessed adequately” to tachycardia and diastolic function unless the algorithm regards shallow syntax which may suppress the false assignment because the value expression is not part of the prepositional phrase of the attribute. We used simple prepositional phrase detection rules for the echocardiography reports and suppressed value assignments between attributes inside of a detected prepositional phrase and values outside of it. As a result, the system is able to avoid certain kinds of false positive extractions based on shallow syntactic structure detection. Full syntactic parsing, for example, using a dependency parser, can be integrated for domains with more complex sentence structure.

Subsequent to document structure detection, the attribute-value matching module iterates over subsections, sentences and segments of the document. For each segment, it first detects objects and attribute candidates and for each attribute possible values. If the type of the attribute and value candidates allows extraction without disambiguation, they are accepted directly, otherwise the algorithm searches for appropriate objects. In a nutshell, objects inside the same segment are preferred, and the scope of the search does not exceed the limits of the containing subsection.

**Fig. 5:**
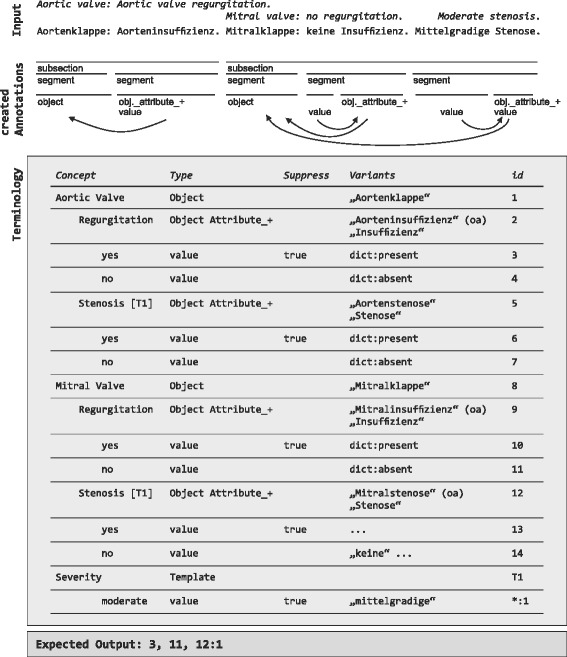
Simplified processing. From top to bottom: input (English, German), subsection annotations, segment annotations, two layers of concept annotations, concept attachments, example terminology. The segmentation algorithm detects subsections and segments. Ambiguity of attributes like stenosis is resolved according to the recognized structure. Postprocessing operations add implicit states for attributes with boolean nature (“Aorteninsuffizienz”, “Stenose”) and remove values that should be suppressed, for instance, at “Insuffizienz”. Suppression activating values are values where the property “suppress” is not true. The expected output contains one value (moderate mitral valve stenosis) that has been imported to the attribute mitral valve stenosis (id=12) from template T1 (Severity). Their ids are composed to 12:1

In order to allow basic semantic postprocessing, there are a few special properties of terminology concepts that further influence extraction behaviour. For example, attributes without value extractions are meaningless unless they are tagged as attributes that have a boolean nature. In this case, they get an implicit state that signals the presence of the attribute. This value is inferred by default, however, it may be suppressed. This is because the terminology declares suppression relations for all value concepts to handle negation. When a suppression activator is found during processing, all other detected passive values of the corresponding attribute are rejected. Consider the example given in Fig. 5. In the 4th segment annotation from the left (“no regurgitation”), the value that indicates presence of mitral valve regurgitation is suppressed because a suppression activating value has been found. On the contrary, moderate mitral valve stenosis and its presence value are not suppressed because no activator has been found here.

As noted in the process model, specific adaptations can be made for each project. For instance, many kinds of clinical reports contain sections that should be ignored. For this reason, the preprocessing scripts allow to focus processing on certain parts of the document. In this work, we recognized that some reports contained multiple examinations, hence, we extended the preprocessing rules in order to detect and ignore subsections that refer to transesophageal echocardiographams (TEE) as Example 7 in Fig. 1.

### Mapping to a standardized guideline

While terminologies created from data suffice for many applications, mappings to standardized guidelines promote portability and help on quality control. General purpose clinical terminologies like SNOMED CT [9] or LOINC [11] cover different aspects of echocardiography reports and can be used for interoperability. However, we chose a different basis for our work. A number of medical societies developed special recommendations for the structure of echocardiography reports, hence, summarizing an experts’ choice of appropriate concepts of this field. As part of this study, we created a mapping between the constructed terminology and the central elements as defined in a guideline for German transthoracic echocardiography reports [18] (core set of parameters), which will be called *Θ*_core_ in the following. The original list contains 28 principal parameters. Some of them have internal structure and represent multiple attributes. In this work, we excluded general information (8 parameters) like patient identification or day of birth but considered internal structure which led to 29 central aspects (cf. Table 6). For all of them, we found associated attributes in the terminology. However, mapping the internal structure accurately was difficult in some cases, especially, valve morphology.

### Ethical approval

The research addressed in this paper did not comprise a patient study, but the extraction of structured data from unstructured echocardiography reports in the clinical data warehouse of the University Hospital of Würzburg. Only pseudonymized patient data was used and patient IDs were neither required nor used for this work. The installation and operation of the clinical data warehouse has been approved by the center for data security (oberster Datenschutzbeauftragter) of the University Hospital of Würzburg.

## Results and discussion

### Setting

The terminology has mainly been curated by one person (HC). Terminology construction and adaptation of segmentation rules was predominantly based on 520 training documents, named *Dev*. Sampling of this set was slightly biased towards more recent documents. Inside of *Dev*, two dominant layout styles (A, B) influenced most of the reports. However, there were also 6 reports that belonged to a third layout style (C), and some unstructured or exceptionally short reports.

We recognized that documents with layout C had subsections with a list element layout like “- AK: … - MK: …” while other documents did not. Unstructured reports were typically shorter than semi-structured reports.

Our main interests in this study were two-fold. On the one hand, we were interested in the coverage and in the quality of extractions according to the core set of parameters as defined in *Θ*_core_. On the other hand, we aimed to assess the reliability of the information extraction application according to the different types of document structure.

In order to find appropriate filtering parameters that categorize these documents into the classes *dominant* layout, *uncommon* layout (layout C), and mostly *unstructured* (short), we analysed the distributions of non-whitespace characters, the number of matches against a simple regular expression that detects list elements (lines that begin with a hyphen), and meta data of the reports (their organizational unit/site). We arrived at the filtering settings displayed in Table 4. Less than 5 % of all reports stemmed from one of three sites that were excluded or had less than 100 non-whitespace characters and were mostly defective. These documents were rejected and did not participate in any further categorization. More than 90 % of all reports were covered by a filter that requires at least 800 non-whitespace characters and assumes less than 5 list elements. This set (*T*_d_) was assumed to correspond to reports that conform to the predominant layouts. The filter that was chosen to detect the uncommon layout C matched on 1.5 % of the documents (*T*_c_) and required at least 5 list elements. In order to find unstructured or exceptionally short reports, approximately 4 % of all reports were covered by *T*_u_. These documents had at least 100 but less than 800 non-whitespace characters.

**Table 4:** Corpus statistics

Name	Description	Filter	#	%
	all TTE reports		70441	100.0
	only relevant sites	*f*_site_	68915	97.8
*T*_d_	dominant layouts	*f*_site_, *f*_char≥800_, $\bar {f}_{\text {li}}$	63489	90.1
*T*_u_	mostly unstructured	*f*_site_, *f*_char≥100_, *f*_char<800_, $\bar {f}_{\text {li}}$	2712	3.9
*T*_c_	uncommon layout	*f*_site_, *f*_li_	1041	1.5
	mostly defective	*f*_site_, *f*_char<100_	1673	2.4

*f*_site_: filter that excludes three sites of the hospital. *f*_char≥*n*_: require at least *n* non white space characters. *f*_li_: at least 5 list elements

For all evaluations, matching annotations had to agree in their type, as well as their begin and end offsets. *c*_tp_ denotes the number of matching attribute-value extractions. *c*_fp_ is the number of false positive extractions (human annotator rejected these items), and *c*_fn,covered_ is the number of attribute-value pairs that were not automatically extracted by the system but which were part of the terminology. We were also interested in the coverage of the terminology according to all information that was stated in the test documents. For this reason, human annotators were instructed to measure the amount of mentions of concepts that were not already handled in the terminology (*c*_fn,missing_), e.g., measurements that did not occur in the training data. Given that *c*_fn_=*c*_fn,covered_+*c*_fn,missing_, we computed precision $p = \frac {c_{\text {tp}}}{c_{\text {tp}} + c_{\text {fp}}}$, recall $r = \frac {c_{\text {tp}}}{c_{\text {tp}} + c_{\text {fn}}}$, and $f_{1} = \frac {2 p r}{p + r} $.

In addition to these micro-averages, we also provide macro-averages for aspects belonging to the core set of parameters *Θ*_core_. These metrics average over a set of precision, recall, and *f*_1_ values, respectively.

From the data sets *T*_d_, *T*_c_, *T*_u_ we randomly sampled 100, 20, 20 documents, respectively, for testing. Each of these sets was then annotated semi-automatically by two different annotators. We assessed their agreement in terms of accuracy on attribute-value pair annotations. Annotations matched if they had the same type, begin and end offsets. Agreement on documents with standard layouts was 95.5 %. Agreement on the uncommon layout and on short documents was lower (*T*_c_: 86.6 %, *T*_u_: 61.1 %). Differences in annotation mostly affected recall. Especially on the data set with short and unstructured reports, one of the annotators tended to create missing attribute annotations while the other annotator found appropriate concepts in the terminology.

After manual inspection, two different investigators decided to choose the annotations of one of the annotators to become the reference standard for the comparison against the automatically extracted information. This annotator was more familiar with the terminology, detected more errors, and created annotations against the terminology more accurately.

On the one hand, system performance was evaluated according to the different types of document structure. On the other hand, we evaluated the performance of attributes that were covered by the core set of parameters as defined in *Θ*_core_.

Please note that previous work contributed to the initially available terms and terminology refinement. The corresponding terminology was constructed from analyzing about 1000 documents from the total set of about 70,000 reports. Unfortunately, we were not able to identify these documents. However, the chance that, for instance, a randomly selected test set of size 100 from the 70,000 documents has an overlap with these 1000 documents is just $ \frac {1000}{70000} \cdot 100 $, i.e. just 1 or 2 documents. Moreover, documents outside of *Dev* have not been handled adequately and gained less attention. They can be considered as an orientation set. Note that, more importantly, the collection *Dev* and the test sets are disjoint.

### Results

Table 5 lists the performance of the information extraction application on the different kinds of document categories. The category that represented 90 % of the whole corpus was processed best with a micro-averaged *f*_1_ score of.978. The precision was.996 and recall.961.

**Table 5:** Results on different kinds of corpora. *α*: fraction of documents belonging to this category

Corpus	# Test doc.	*α*	tp	fp	fn	Precision	Recall	*f*_1_
*T*_d_	100	.90	5332	23	214	.996	.961	.978
*T*_c_	20	.02	730	19	116	.975	.863	.915
*T*_u_	20	.04	126	11	99	.920	.560	.696

Documents having the uncommon layout (C) were handled less accurate (*f*_1_=.915). While extractions made by the system remained very accurate on these documents (precision.975), recall fell by.098 (absolute) to.863. A further decrease in performance was measured on the third category of documents (exceptionally short and unstructured reports): *f*_1_=.696. Again, precision (.920) decreased less ($\Delta ^{\text {prec.}}_{\text {d,u}}=.058$) than recall (*r*_u_=.560, $\Delta ^{\text {rec.}}_{\text {d,u}}=.401$).

Results regarding the core set of parameters *Θ*_core_ are shown in Table 6. Recognition of attributes and values was performed considerably better than on average (micro averaged *f*_1_=.989, precision =.993, recall =.986). Only 20 out of 2892 extractions were wrong. There were three main sources of these errors. Six errors were caused by negation or insufficient handling of prepositional phrases. In five cases concepts or variants were not specified properly. The remaining false positives were due to missing concepts or variants so that existing but wrong concepts were extracted. There were 41 false negatives, that is, manually annotated attribute value pairs that were not recognized automatically by the system. Most of them were caused by missing variants or concepts. Fifteen false negatives addressed aortic valve morphology, especially the mobility of the aortic valve.

Notably, the macro averages that were achieved across the 29 parameters shown in Table 6 are very high: precision =.99, recall =.95, *f*_1_=.96. That is, the system performs well on each of these aspects in general. Exceptions were some infrequent items, for instance, mitral stenosis, which only occurred three times in the test set.

**Table 6:** Results of information extraction according to 29 important aspects of transthoracic echocardiography reports, measured on 100 test documents of category *T*_d_

Section	Aspect	TP	FP	FN	Prec	Rec.	F1
General Information							
	Prosthetic Valves (type)	16	0	3	1.00	0.84	0.91
	Imaging Quality	263	0	3	1.00	0.99	0.99
	No Regurgitation	8	0	0	1.00	1.00	1.00
Aortic Valve							
	Morphology	74	0	15	1.00	.83	.91
	V _max_	84	0	0	1.00	1.00	1.00
	AV Regurgitation	136	1	0	.99	1.00	1.00
	AV Prosthesis Regurgitation	8	0	0	1.00	1.00	1.00
	AV Stenosis	48	0	1	1.00	.98	.99
	AV Area	15	2	0	.88	1.00	.94
	*ΔP*_max_ (pressure gradient)	23	2	0	.92	1.00	.96
Aorta	Aortic Root Diameter	186	0	0	1.00	1.00	1.00
Mitral Valve							
	Morphology	3	0	0	1.00	1.00	1.00
	MV Regurgitation	229	6	1	.97	1.00	.98
	MV Prosthesis (Pmean, PHT)	4	0	2	1.00	.67	.80
	MV Stenosis	1	0	2	1.0	.33	.50
	MV Area	2	0	0	1.00	1.00	1.00
Tricuspid Valve							
	Morphology	67	0	0	1.00	1.00	1.00
	TV Regurgitation	259	2	0	.99	1.00	1.00
	sPAP	178	0	0	1.00	1.00	1.00
Left Ventricle							
	LVD _d_	15	0	0	1.00	1.00	1.00
	LVD _s_	67	0	0	1.00	1.00	1.00
	IVSD _d_	97	0	0	1.00	1.00	1.00
	PWD _d_	96	0	1	1.00	.99	.99
	LVEF (%)	221	0	0	1.00	1.00	1.00
	Wall Motion Abnormalities	51	0	1	1.00	.98	.99
	Diastolic Function	350	2	6	.99	.98	.99
Left Atrium	LAD (LADs, LADsI)	88	0	0	1.00	1.00	1.00
Right Ventricle	Dimension	101	0	1	1.00	.99	.99
Pericardium	Pericardial Effusion	182	5	5	.97	.97	.97
Micro Average		2872	20	41	.993	.986	.989
Macro Average	(mean over all aspects)				.991	.950	.963

Several attribute-value pairs may belong to each aspect, for instance, absolute and relative mentions

### Discussion

In summary, the system performed very well. Especially information that belongs to the core set of parameters was extracted with *f*_1_=.96 (macro average), =.99 (micro average). Hence, the system supports the central aspects of echocardiography reports. Recognition rates with respect to all types of information are only slightly lower (micro-averaged *f*_1_=.978). These figures apply to the majority (90 *%*) of the whole set of TTE reports of the University Hospital of Würzburg. The detailed categorization of documents revealed that semi-structured reports with an uncommon layout were processed less accurate but also fairly well (micro avg. *f*_1_=.915). In particular, the information extraction system obtains high precision (micro avg..920) even on unstructured or exceptionally short reports. Hence, the entity disambiguation algorithm of the current system works very well while the generalization capabilities of the term recognition module leave room for improvements; this work applied adapted key word matching which shall be relaxed in other domains. It had only minor implications in this project, because most documents stemmed from a few predominant layouts that we were able to categorize with appropriate filtering. Therefore, the majority of documents was processed accurately.

Finally, the following limitations of our approach remain to be addressed in the future. Although the terminology contains a broad range of concepts and terms, the slight sampling bias towards more recent years and the relatively small sampling size might have caused a limited coverage of terms of older documents or very rare, but important pathological findings. For each document, the majority of concepts is covered but it is unclear how many distinct concepts were missed. Put in different words, our results shed light on the general performance of the information extraction algorithm, especially, on term recognition and disambiguation. Subsequent studies are however necessary to analyse the comprehensiveness of the terminology.

Changes to the current system and its knowledge representation with object-attribute-value structures and templates that only accept values may be required to handle temporal or spatial relations correctly.

## Conclusions

In the past, ontology-driven rule-based systems have shown very good results for information extraction in several clinical domains, however, this process is known to be time-consuming and costly. As a consequence, there is a lack of such components for many languages and domains.

This work addressed information extraction from German transthoracic echocardiography reports. Data-driven development with special tools produced a fine-grained terminology with a broad set of parameters. The final system achieves state-of-the-art precision (.996 micro average) and recall (.961 micro average), *f*_1_=.978, on the majority of documents of the University Hospital of Würzburg. It covers the central standardized aspects of the domain, which have even better recognition rates (micro avg. *f*_1_=.99, macro avg. *f*_1_=.96). In order to provide more detailed information about the quality of extractions for users of the data, we measured performance on different kinds of categories of reports. Based on simple assumptions on document structure, we assessed different sets of documents where we assumed less accurate output. Empirical results were in line with our expectations. Uncommon semi-structured reports were processed slightly less accurate than documents with the standard layouts. Notably, precision remained on a high level even on unstructured and exceptionally short reports.

The systems and tools that facilitated this study are currently in use for building information extraction applications for other kinds of clinical reports with noun phrases, among others: electrocardiography, physical examination, or lung function tests. A major challenge is the extraction of information from complex sentences. At the University Hospital of Würzburg, this constitutes a small but relevant amount of information, for instance, in the domains patient history or epicrisis. While simple rules detected and attached prepositional phrases in this study on semi-structured echocardiography reports, we have conducted first experiments to integrate a dependency parser for enhanced performance. Further challenges are the resolution of temporal expressions and the correct interpretation of intentionally vague indications of degrees of certainty that appear in some reports.

Moreover, we intend to intensify our work on quality estimation based on background knowledge. In future work, we will use medical background knowledge for constrained-driven evaluation of the extracted information.

## Additional file

Additional file 1 **HTML-formatted TTE terminology.** (HTML 117 kb)
