# Erratum to: Fine-grained information extraction from German transthoracic echocardiography reports

**DOI:** 10.1186/s12911-015-0226-7

**Published:** 2015-12-03

**Authors:** Martin Toepfer, Hamo Corovic, Georg Fette, Peter Klügl, Stefan Störk, Frank Puppe

**Affiliations:** Chair of Computer Science VI, University of Würzburg, Am Hubland, Würzburg, D-97074 Germany; Comprehensive Heart Failure Center, University of Würzburg, Straubmühlweg 2a, Würzburg, D-97078 Germany; Averbis GmbH, Tennenbacher Straße 11, Freiburg, D-79106 Germany

After publication of the original article [1] it was brought to our attention that figures three and four had been interchanged on the website, meaning that they were then matched with the wrong figure legends. This has now been updated on the website.
