# Supplementary material for: Fine-grained information extraction from German transthoracic echocardiography reports
Source: BMC Med Inform Decis Mak. 2015 Nov 12;15:91. doi: 10.1186/s12911-015-0215-x (PMC4643516; doi:10.1186/s12911-015-0215-x)
Supplement: Additional file 1 — HTML-formatted TTE terminology. (HTML 117 kb) [file 12911_2015_215_MOESM1_ESM.html]

# Echo TTE

### Table of Contents

1. Concepts
2. Templates

### Concepts

- weitere Messwerte
  [
  structure
  , id=
  765
  ]
  - Echogerät:
    [
    attribute
    , id=
    616
    ]
    - Vivid Seven
      [
      bool
      , id=
      617
      ]
  - ZVD
    [
    attribute
    , id=
    756
    , templates="
    Messung\_mmHg
    "
    ]
    Central Venous Pressure
  - Septum
    [
    attribute
    , id=
    845
    , templates="
    Messung\_mm
    "
    ]
    Muskelwand, die die beiden Ventrikel trennt
  - Hinterwand
    [
    attribute
    , id=
    847
    , templates="
    Messung\_mm
    "
    ]
  - TD-E'
    [
    attribute
    , id=
    979
    , templates="
    Messung\_cm\_per\_s
    "
    ]
    tissue doppler derived E'
  - BSA
    [
    attribute
    , id=
    985
    , templates="
    Messung\_qm
    "
    ]
    Körperoberfläche
  - FS
    [
    attribute
    , id=
    1200
    , templates="
    Messwert\_Prozent
    "
    ]
    Fractional shortening
  - SVI
    [
    attribute
    , id=
    1290
    , templates="
    Messung\_ml\_per\_qm
    "
    ]
    Stroke Volume
  - RWD
    [
    attribute
    , id=
    1322
    ]
    right wall diameter
    - Wert
      [
      numeric\_eq
      , id=
      1372
      ]
  - Flow
    [
    attribute
    , id=
    1364
    , templates="
    Messung
    "
    ]
  - RR
    [
    attribute
    , id=
    1398
    , templates="
    Messung\_mmHg
    "
    ]
    Blood Pressure
  - Sinus
    [
    attribute
    , id=
    1408
    , templates="
    Messung\_mm
    "
    ]
    Vene der Herzmuskulatur
  - VCI-exsp
    [
    attribute
    , id=
    1410
    , templates="
    Messung\_mm
    "
    ]
    Exspiratory Vena Cava Inferior Diameter
  - RADs-I
    [
    attribute
    , id=
    1413
    , templates="
    Messung\_m\_pro\_mm2
    "
    ]
    right wall diameter septal-lateral
  - RV/RA-Grad.
    [
    attribute
    , id=
    1418
    , templates="
    Messung\_mmHg
    "
    ]
    Right Ventricular/Right Atrial Gradient
  - Aorta
    [
    structure
    , id=
    1546
    ]
    - Ao\_ST
      [
      attribute
      , id=
      1409
      , templates="
      Messung\_mm
      "
      ]
    - Ao-asc
      [
      attribute
      , id=
      932
      , templates="
      Messung\_mm
      "
      ]
      Ascending Aortic Diameter = Durchmesser der Aorta ascendens
      - ektatisch
        [
        bool
        , id=
        1211
        ]
      - Normal
        [
        bool
        , id=
        1214
        ]
      - aneurysmatisch
        [
        bool
        , id=
        1383
        ]
    - Ao-Ring
      [
      attribute
      , id=
      1407
      , templates="
      Messung\_mm
      "
      ]
      Aortenring
  - Aortenklappe
    [
    structure
    , id=
    1547
    ]
    - AI-PHT
      [
      attribute
      , id=
      977
      , templates="
      Messung\_ms
      "
      ]
      Aortenklappeninsuffizienz-Druckhalbwertszeit
    - AI-VC
      [
      attribute
      , id=
      1266
      , templates="
      Messung\_mm
      "
      ]
      Aorteninsuffizienz - Valve Closure
    - AR-PHT
      [
      attribute
      , id=
      951
      , templates="
      Messung\_ms
      "
      ]
      Aortic Regurgitation Pressure Half-Time = Aorteninsuffizienz Druckhalbwertszeit
    - AR-proxD/LVOT
      [
      attribute
      , id=
      1299
      , templates="
      Messwert\_Prozent
      "
      ]
      aortic regurgitation-proximal diameter/left ventricular outflow tract
    - AVA
      [
      attribute
      , id=
      1280
      , templates="
      Messung\_qcm
      "
      ]
      Aortic Valve Area
    - AV-VTI
      [
      attribute
      , id=
      632
      , templates="
      Messung\_cm
      "
      ]
      Aortic Valve Velocity Time Integral
  - AR-VC
    [
    attribute
    , id=
    1390
    , templates="
    Messung\_mm
    "
    ]
    Arrhythmogene rechtsventrikuläre Kardiomyopathie
  - Linker Ventrikel
    [
    structure
    , id=
    1548
    ]
    - EDVI
      [
      attribute
      , id=
      1286
      , templates="
      Messung\_ml\_per\_qm
      "
      ]
      enddiastolischer Volumenindex des linken Ventrikels
    - ESVI
      [
      attribute
      , id=
      1288
      , templates="
      Messung\_ml\_per\_qm
      "
      ]
      endosystolischer Volumenindex des linken Ventrikels
    - LVDdI
      [
      attribute
      , id=
      955
      ]
      Left Ventricular Diameter, diastolic
      - Messung mm/m²
        [
        numeric\_eq
        , id=
        956
        ]
    - LVDsI
      [
      attribute
      , id=
      953
      ]
      Left Ventricular Diameter, systolic
      - Messung mm/m²
        [
        numeric\_eq
        , id=
        954
        ]
    - LVFS
      [
      attribute
      , id=
      625
      , templates="
      Messwert\_Prozent
      "
      ]
      Left Ventricular Fractional Shortening
    - LVOT
      [
      attribute
      , id=
      1297
      , templates="
      Messung\_mm
      "
      ]
      Left Ventricular Outflow Tract
    - LVOT-Pmax
      [
      attribute
      , id=
      1391
      , templates="
      Messung\_mmHg
      "
      ]
      Left Ventricular Outflow Tract Peak Pressure
    - LVOT-Vmax
      [
      attribute
      , id=
      974
      , templates="
      Messung\_m\_per\_s
      "
      ]
      Left Ventricular Outflow Peak Velocity
    - LVOT-VTI
      [
      attribute
      , id=
      965
      , templates="
      Messung\_cm
      "
      ]
      Left Ventricular Outflow Tract Velocity Time Integral
    - IVRT
      [
      attribute
      , id=
      900
      , templates="
      Messung\_ms
      "
      ]
      Isovolumic Relaxation Time = Isovolumetrische Relaxationszeit
    - Vmax A-Welle
      [
      attribute
      , id=
      1576
      , templates="
      Messung\_m\_per\_s Default
      "
      ]
    - Vmax E-Welle
      [
      attribute
      , id=
      1577
      , templates="
      Messung\_m\_per\_s Default
      "
      ]
  - Trikuspidalklappe
    [
    structure
    , id=
    1549
    ]
    - TAPSE
      [
      attribute
      , id=
      917
      , templates="
      Messung\_mm
      "
      ]
      Tricuspid Annular Plane Systolic Excursion
    - TR-VC
      [
      attribute
      , id=
      1397
      , templates="
      Messung\_mm
      "
      ]
      tricuspid regurgitation - valve closure
    - TR-Vmax
      [
      attribute
      , id=
      639
      , templates="
      Messung\_m\_per\_s
      "
      ]
      Tricuspid Regurgitation Peak Velocity
    - TV-Pmax
      [
      attribute
      , id=
      1578
      , templates="
      Default Messung\_mmHg
      "
      ]
      Trikuspidalklappe maximaler Druck
    - TI-Vmax
      [
      attribute
      , id=
      1579
      , templates="
      Messung\_m\_per\_s Default
      "
      ]
      Trikuspidalinsuffizienz max Geschwindigkeit
  - Mitralklappe
    [
    structure
    , id=
    1550
    ]
    - MAPSE lat.
      [
      attribute
      , id=
      1393
      , templates="
      Messung\_cm
      "
      ]
      Mitral Annular Plane Systolic Excursion, lateral
    - MAPSE sept.
      [
      attribute
      , id=
      1392
      , templates="
      Messung\_cm
      "
      ]
      Mitral Annular Plane Systolic Excursion, septal
    - MI-VC
      [
      structure
      , id=
      1264
      ]
      Mitralinsuffizienz - valve closure
    - M-Ring-E'
      [
      attribute
      , id=
      637
      , templates="
      Messung\_cm\_per\_s
      "
      ]
      mitral ring E'
    - MR-RV
      [
      attribute
      , id=
      1396
      , templates="
      Messung\_ml
      "
      ]
      Mitral Regurgitation: Regurgitant Volume Flow
    - MR-VC
      [
      attribute
      , id=
      1244
      , templates="
      Messung\_mm
      "
      ]
      mitral regurgitation – valve closure
    - MV-A
      [
      attribute
      , id=
      634
      , templates="
      Messung\_m\_per\_s
      "
      ]
      MV Velocity Peak A
    - MV-Adur
      [
      attribute
      , id=
      959
      , templates="
      Messung\_ms
      "
      ]
      Mitral Valve A-Wave Duration
    - MV-A-Dur/PV-A-Dur
      [
      attribute
      , id=
      961
      ]
      Mitral Valve A-Wave Duration/Pulmonic Valve A-Wave Duration
      - Verhältnis
        [
        numeric\_eq
        , id=
        962
        ]
    - MVA-PHT
      [
      attribute
      , id=
      1369
      , templates="
      Messung\_qcm
      "
      ]
      Mitral Valve Area Pressure Half Time
    - MV-E
      [
      attribute
      , id=
      633
      , templates="
      Messung\_m\_per\_s
      "
      ]
      Mitral Valve Velocity Peak E
    - MV-E/A
      [
      attribute
      , id=
      635
      ]
      Mitral Valve E-Peak to A-Peak Ratio
      - Verhältnis
        [
        numeric\_eq
        , id=
        656
        ]
    - MV-ERO
      [
      attribute
      , id=
      957
      ]
      Mitral Valve Effective Regurgitant Orifice
      - Messung cm²
        [
        numeric\_eq
        , id=
        958
        ]
    - MV-PHT
      [
      attribute
      , id=
      1366
      , templates="
      Messung\_ms
      "
      ]
      Mitral Valve Pressure Half Time
    - MV-Pmean
      [
      attribute
      , id=
      1367
      , templates="
      Messung\_mmHg
      "
      ]
      Mitral Valve Mean Pressure
    - MV-Vmax
      [
      attribute
      , id=
      1368
      , templates="
      Messung\_m\_per\_s
      "
      ]
      Mitral Valve Peak Velocity
    - MV-VTI
      [
      attribute
      , id=
      1295
      , templates="
      Messung\_cm
      "
      ]
      Mitral Valve Velocity Time Integral
    - DT (MV-E)
      [
      attribute
      , id=
      636
      , templates="
      Messung\_ms
      "
      ]
      Deceleration Time (Mitral Valve Velocity Peak E)
    - E/E'
      [
      attribute
      , id=
      638
      ]
      Verhältnis zwischen der maximalen Geschwindigkeiten des passiven Mitraleinstromprofils (E) und der lateralen frühdiastolischen Mitralanulusgeschwindigkeit (E')
      - Verhältnis
        [
        numeric\_eq
        , id=
        659
        ]
  - Rechter Vorhof
    [
    structure
    , id=
    1551
    ]
    - RA-Area
      [
      attribute
      , id=
      1389
      , templates="
      Messung\_qcm
      "
      ]
      Right Atrium Area
    - RAP
      [
      attribute
      , id=
      1343
      , templates="
      Messung\_mmHg
      "
      ]
      Right Atrial Pressure
  - Linker Vorhof
    [
    structure
    , id=
    1552
    ]
    - LA\_pl
      [
      attribute
      , id=
      1406
      , templates="
      Messung\_qcm
      "
      ]
    - LA-area
      [
      attribute
      , id=
      914
      ]
      Left Atrium Area
      - Messung in cm²
        [
        numeric\_eq
        , id=
        915
        ]
  - Pulmonalklappe
    [
    structure
    , id=
    1553
    ]
    - PV-A-Dur
      [
      attribute
      , id=
      1400
      , templates="
      Messung\_ms
      "
      ]
      Pulmonic Valve A-Wave Duration
    - PV-VTI
      [
      attribute
      , id=
      1293
      , templates="
      Messung\_cm
      "
      ]
      Pulmonic Valve Velocity Time Integral
  - Pulmonalarterie
    [
    structure
    , id=
    1554
    ]
    - PA-Druck
      [
      attribute
      , id=
      1216
      , templates="
      Messung\_mmHg
      "
      ]
      Pulmonary Artery Pressure
  - Rechter Ventrikel
    [
    structure
    , id=
    1555
    ]
    - RVD
      [
      attribute
      , id=
      1395
      , templates="
      Messung\_mm
      "
      ]
      right ventricular diameter
    - RVESP
      [
      attribute\_plus
      , id=
      1440
      , templates="
      Messung\_mmHg
      "
      ]
      right ventricular endsystolic pressure
      - negiert
        [
        bool
        , id=
        1441
        ]
      - vorhanden
        [
        bool
        , id=
        1442
        ]
      - erhöht
        [
        bool
        , id=
        1444
        ]
    - RVOT9
      [
      attribute
      , id=
      1405
      , templates="
      Messung\_mm
      "
      ]
      Right Ventricle Outflow Tract
- Beurteilung
  [
  structure
  , id=
  766
  ]
  - Transthorakale Echokardiographie
    [
    attribute\_plus
    , id=
    662
    ]
    - Datum
      [
      bool
      , id=
      767
      ]
    - negiert
      [
      bool
      , id=
      1247
      ]
    - vorhanden
      [
      bool
      , id=
      1248
      ]
  - Sinusrhythmus
    [
    attribute\_plus
    , id=
    670
    ]
    - negiert
      [
      bool
      , id=
      1167
      ]
    - vorhanden
      [
      bool
      , id=
      1168
      ]
  - Herzfrequenz
    [
    attribute
    , id=
    671
    ]
    - Messwert (pro Min.)
      [
      numeric\_eq
      , id=
      672
      ]
    - Intervall untere Grenze
      [
      numeric\_eq
      , id=
      774
      ]
    - hyperkinetisch
      [
      bool
      , id=
      1436
      ]
  - Wandbewegungsstörungen
    [
    attribute\_plus
    , id=
    729
    ]
    - linksventrikulär
      [
      bool
      , id=
      731
      ]
    - rechtsventrikulär
      [
      bool
      , id=
      826
      ]
    - regional
      [
      bool
      , id=
      990
      ]
    - nicht beurteilbar
      [
      bool
      , id=
      1023
      ]
    - negiert
      [
      bool
      , id=
      1306
      ]
    - vorhanden
      [
      bool
      , id=
      1307
      ]
  - rechtsventrikuläre systolische Funktion
    [
    attribute
    , id=
    735
    , templates="
    Systolische\_Funktion
    "
    ]
  - Pathologie
    [
    structure
    , id=
    741
    ]
    - Linksherzhypertrophie
      [
      attribute\_plus
      , id=
      867
      , templates="
      Schweregrade
      "
      ]
      - negiert
        [
        bool
        , id=
        1175
        ]
      - vorhanden
        [
        bool
        , id=
        1176
        ]
      - exzentrisch
        [
        bool
        , id=
        869
        ]
      - konzentrisch
        [
        bool
        , id=
        876
        ]
      - nicht beurteilbar
        [
        bool
        , id=
        1151
        ]
    - Lebervenenstau
      [
      attribute\_plus
      , id=
      880
      , templates="
      Default Schweregrade
      "
      ]
      - negiert
        [
        bool
        , id=
        1208
        ]
      - vorhanden
        [
        bool
        , id=
        1209
        ]
    - kardiale Amyloidose
      [
      attribute\_plus
      , id=
      897
      ]
      - negiert
        [
        bool
        , id=
        1179
        ]
      - vorhanden
        [
        bool
        , id=
        1180
        ]
    - Arrythmie
      [
      attribute\_plus
      , id=
      1315
      ]
      - negiert
        [
        bool
        , id=
        1316
        ]
      - vorhanden
        [
        bool
        , id=
        1317
        ]
      - Vorhofflimmern
        [
        attribute\_plus
        , id=
        889
        ]
        - chronisch
          [
          bool
          , id=
          890
          ]
        - negiert
          [
          bool
          , id=
          1228
          ]
        - vorhanden
          [
          bool
          , id=
          1229
          ]
        - nicht beurteilbar
          [
          bool
          , id=
          1310
          ]
      - nicht beurteilbar
        [
        bool
        , id=
        1318
        ]
    - Remodelling
      [
      attribute\_plus
      , id=
      1324
      , templates="
      Hoehle
      "
      ]
      - negiert
        [
        bool
        , id=
        1325
        ]
      - vorhanden
        [
        bool
        , id=
        1326
        ]
      - konzentrisch
        [
        bool
        , id=
        1570
        ]
      - linksventrikulär
        [
        bool
        , id=
        1571
        ]
    - Linksherzinsuffizienz
      [
      attribute\_plus
      , id=
      1327
      ]
      - negiert
        [
        bool
        , id=
        1328
        ]
      - vorhanden
        [
        bool
        , id=
        1329
        ]
    - Bioprothese
      [
      attribute\_plus
      , id=
      1338
      ]
      - negiert
        [
        bool
        , id=
        1339
        ]
      - vorhanden
        [
        bool
        , id=
        1340
        ]
      - in Aortenposition
        [
        bool
        , id=
        1422
        ]
      - in Mitralposition
        [
        bool
        , id=
        1423
        ]
      - normale Prothesenfunktion
        [
        bool
        , id=
        1566
        ]
    - AP-Symptomatik
      [
      attribute\_plus
      , id=
      1345
      ]
      - negiert
        [
        bool
        , id=
        1346
        ]
      - vorhanden
        [
        bool
        , id=
        1347
        ]
    - Tachykardie
      [
      attribute\_plus
      , id=
      1373
      ]
      - negiert
        [
        bool
        , id=
        1374
        ]
      - vorhanden
        [
        bool
        , id=
        1375
        ]
    - Cor hypertonicum
      [
      attribute\_plus
      , id=
      1402
      ]
      - negiert
        [
        bool
        , id=
        1403
        ]
      - vorhanden
        [
        bool
        , id=
        1404
        ]
    - Mechanische Prothese
      [
      attribute\_plus
      , id=
      1419
      ]
      - negiert
        [
        bool
        , id=
        1420
        ]
      - vorhanden
        [
        bool
        , id=
        1421
        ]
      - in Aortenposition
        [
        bool
        , id=
        1424
        ]
      - in Mitralposition
        [
        bool
        , id=
        1425
        ]
    - Hyperkinetische Herzaktion
      [
      attribute\_plus
      , id=
      1427
      ]
      - negiert
        [
        bool
        , id=
        1428
        ]
      - vorhanden
        [
        bool
        , id=
        1429
        ]
    - Pleuraerguss
      [
      attribute\_plus
      , id=
      1430
      ]
      - negiert
        [
        bool
        , id=
        1431
        ]
      - vorhanden
        [
        bool
        , id=
        1432
        ]
      - linksseitig
        [
        bool
        , id=
        1567
        ]
      - rechtsseitig
        [
        bool
        , id=
        1568
        ]
      - beidseitig
        [
        bool
        , id=
        1569
        ]
    - Endokarditis
      [
      attribute\_plus
      , id=
      1433
      ]
      - negiert
        [
        bool
        , id=
        1434
        ]
      - vorhanden
        [
        bool
        , id=
        1435
        ]
    - Septumbewegung
      [
      attribute
      , id=
      1438
      ]
      - paradox
        [
        bool
        , id=
        1439
        ]
    - Infarktnarbe
      [
      attribute\_plus
      , id=
      1445
      ]
      - negiert
        [
        bool
        , id=
        1446
        ]
      - vorhanden
        [
        bool
        , id=
        1447
        ]
    - Asynkronie
      [
      attribute\_plus
      , id=
      1448
      ]
      - negiert
        [
        bool
        , id=
        1449
        ]
      - vorhanden
        [
        bool
        , id=
        1450
        ]
      - linksventrikulär
        [
        bool
        , id=
        1451
        ]
    - Transposition der großen Gefäße
      [
      attribute\_plus
      , id=
      1455
      ]
      - negiert
        [
        bool
        , id=
        1456
        ]
      - vorhanden
        [
        bool
        , id=
        1457
        ]
      - Kongenital korrigiert
        [
        bool
        , id=
        1458
        ]
    - Kardiomyopathie
      [
      attribute\_plus
      , id=
      1459
      ]
      - negiert
        [
        bool
        , id=
        1460
        ]
      - vorhanden
        [
        bool
        , id=
        1461
        ]
      - dilatative
        [
        bool
        , id=
        1462
        ]
      - hypertrophe
        [
        bool
        , id=
        1463
        ]
      - ischämische
        [
        bool
        , id=
        1464
        ]
      - Fabry
        [
        bool
        , id=
        1465
        ]
    - PFO
      [
      attribute\_plus
      , id=
      1466
      ]
      persistierendes Foramen Ovale
      - negiert
        [
        bool
        , id=
        1467
        ]
      - vorhanden
        [
        bool
        , id=
        1468
        ]
    - Vorhofohr
      [
      attribute
      , id=
      1475
      ]
      - frei
        [
        bool
        , id=
        1476
        ]
    - Thromben
      [
      attribute\_plus
      , id=
      1477
      ]
      - negiert
        [
        bool
        , id=
        1478
        ]
      - vorhanden
        [
        bool
        , id=
        1479
        ]
    - Papillarmuskel
      [
      attribute
      , id=
      1480
      ]
      - prominent
        [
        bool
        , id=
        1481
        ]
    - Papillarmuskeldysfunktion
      [
      attribute\_plus
      , id=
      1482
      ]
      - negiert
        [
        bool
        , id=
        1483
        ]
      - vorhanden
        [
        bool
        , id=
        1484
        ]
    - Shunt
      [
      attribute\_plus
      , id=
      1485
      ]
      - negiert
        [
        bool
        , id=
        1486
        ]
      - vorhanden
        [
        bool
        , id=
        1487
        ]
      - auf Vorhofebene
        [
        bool
        , id=
        1488
        ]
    - Myokarditis
      [
      attribute\_plus
      , id=
      1489
      ]
      - negiert
        [
        bool
        , id=
        1490
        ]
      - vorhanden
        [
        bool
        , id=
        1491
        ]
    - LV-Asynchronie
      [
      attribute\_plus
      , id=
      1502
      ]
      - negiert
        [
        bool
        , id=
        1503
        ]
      - vorhanden
        [
        bool
        , id=
        1504
        ]
    - Septumdefekt
      [
      structure
      , id=
      1541
      ]
      - ASD
        [
        attribute\_plus
        , id=
        1469
        ]
        Vorhofseptumdefekt
        - negiert
          [
          bool
          , id=
          1470
          ]
        - vorhanden
          [
          bool
          , id=
          1471
          ]
      - VSD
        [
        attribute\_plus
        , id=
        1472
        ]
        Ventrikelseptumdefekt
        - negiert
          [
          bool
          , id=
          1473
          ]
        - vorhanden
          [
          bool
          , id=
          1474
          ]
  - Herzhöhlen
    [
    attribute
    , id=
    762
    , templates="
    Label\_Anatomie
    "
    ]
    - normal groß
      [
      bool
      , id=
      763
      ]
    - linksseitig
      [
      bool
      , id=
      906
      ]
    - rechtsseitig
      [
      bool
      , id=
      992
      ]
    - nicht beurteilbar
      [
      bool
      , id=
      1157
      ]
    - im übrigen normal
      [
      bool
      , id=
      1382
      ]
  - Klappeninsuffizienzen
    [
    attribute
    , id=
    854
    ]
    - keine
      [
      bool
      , id=
      855
      ]
    - nicht beurteilbar
      [
      bool
      , id=
      1158
      ]
  - Granular sparkling
    [
    attribute\_plus
    , id=
    891
    ]
    - negiert
      [
      bool
      , id=
      1181
      ]
    - vorhanden
      [
      bool
      , id=
      1182
      ]
  - Kontraktionsablauf
    [
    attribute
    , id=
    910
    ]
    - normal
      [
      bool
      , id=
      911
      ]
    - im übrigen normal
      [
      bool
      , id=
      1381
      ]
  - Vorhofseptum
    [
    attribute
    , id=
    919
    ]
    - hypermobil
      [
      bool
      , id=
      920
      ]
    - nicht hypermobil
      [
      bool
      , id=
      1141
      ]
    - nicht beurteilbar
      [
      bool
      , id=
      1160
      ]
    - aneurysmatisch
      [
      bool
      , id=
      1348
      ]
  - Hypertonie
    [
    attribute\_plus
    , id=
    928
    , templates="
    Default Schweregrade
    "
    ]
    - negiert
      [
      bool
      , id=
      1183
      ]
    - vorhanden
      [
      bool
      , id=
      1184
      ]
    - pulmonal
      [
      bool
      , id=
      929
      ]
  - Septumwulst
    [
    attribute\_plus
    , id=
    963
    , templates="
    Default Schweregrade
    "
    ]
    - negiert
      [
      bool
      , id=
      1185
      ]
    - vorhanden
      [
      bool
      , id=
      1186
      ]
    - keine Obstruktion
      [
      bool
      , id=
      964
      ]
    - Messung mm
      [
      numeric\_eq
      , id=
      1018
      ]
  - Mitralklappenreflux
    [
    attribute\_plus
    , id=
    984
    ]
    - negiert
      [
      bool
      , id=
      1187
      ]
    - vorhanden
      [
      bool
      , id=
      1188
      ]
  - Aneurysma
    [
    attribute\_plus
    , id=
    989
    ]
    - negiert
      [
      bool
      , id=
      1191
      ]
    - vorhanden
      [
      bool
      , id=
      1192
      ]
  - Wandbewegung
    [
    attribute
    , id=
    994
    ]
    - nicht beurteilbar
      [
      bool
      , id=
      995
      ]
    - negiert
      [
      bool
      , id=
      1270
      ]
    - vorhanden
      [
      bool
      , id=
      1271
      ]
  - Pulmonalklappe
    [
    object
    , id=
    1011
    ]
    - Insuffizienz
      [
      object\_attribute\_plus
      , id=
      1197
      , templates="
      Insuffizienz
      "
      ]
      - negiert
        [
        bool
        , id=
        1198
        ]
      - vorhanden
        [
        bool
        , id=
        1199
        ]
    - Zustand
      [
      object\_attribute
      , id=
      1235
      ]
      - unauffällig
        [
        bool
        , id=
        759
        ]
      - nicht beurteilbar
        [
        bool
        , id=
        1139
        ]
    - Öffnungsfläche
      [
      object\_attribute
      , id=
      1519
      ]
      - normal
        [
        bool
        , id=
        1520
        ]
      - planimetrisch
        [
        bool
        , id=
        1521
        ]
      - eingeschränkt
        [
        bool
        , id=
        1522
        ]
    - Stenose
      [
      object\_attribute\_plus
      , id=
      1556
      , templates="
      Default Schweregrade
      "
      ]
      - negiert
        [
        bool
        , id=
        1557
        ]
      - vorhanden
        [
        bool
        , id=
        1558
        ]
    - PV-Vmax
      [
      object\_attribute
      , id=
      1572
      , templates="
      Messung\_m\_per\_s Default
      "
      ]
  - Rechtsherzbelastung
    [
    attribute\_plus
    , id=
    1276
    ]
    - negiert
      [
      bool
      , id=
      1277
      ]
    - vorhanden
      [
      bool
      , id=
      1278
      ]
    - akut
      [
      bool
      , id=
      1279
      ]
    - chronisch
      [
      bool
      , id=
      1573
      ]
  - Prothesenfunktion
    [
    attribute
    , id=
    1330
    ]
    - normal
      [
      bool
      , id=
      1331
      ]
    - negiert
      [
      bool
      , id=
      1335
      ]
    - vorhanden
      [
      bool
      , id=
      1336
      ]
    - pathologisch
      [
      bool
      , id=
      1496
      ]
  - Rechter Vorhof
    [
    object
    , id=
    1559
    ]
    - R. Vorhof (Zustand)
      [
      object\_attribute
      , id=
      894
      , templates="
      Vorhof
      "
      ]
- Herzklappen allgemein
  [
  structure
  , id=
  903
  ]
  - Flussgeschwindigkeit über allen Herzklappen
    [
    attribute
    , id=
    760
    ]
    - normal
      [
      bool
      , id=
      761
      ]
  - Herzklappen Zustand
    [
    attribute
    , id=
    1233
    ]
    - nicht beurteilbar
      [
      bool
      , id=
      1165
      ]
    - unauffällig
      [
      bool
      , id=
      904
      ]
    - im übrigen normal
      [
      bool
      , id=
      1380
      ]
  - TAVI
    [
    attribute\_plus
    , id=
    1499
    ]
    Aortenklappenersatz
    - negiert
      [
      bool
      , id=
      1500
      ]
    - vorhanden
      [
      bool
      , id=
      1501
      ]
- Zusatzinformationen
  [
  structure
  , id=
  1527
  ]
  - Annotation fehlt
    [
    attribute
    , id=
    991
    ]
    - AnnotationFehlt
      [
      bool
      , id=
      1272
      ]
    - Annotation Wert fehlt
      [
      bool
      , id=
      1282
      ]
  - Dyspnoe
    [
    attribute\_plus
    , id=
    982
    ]
    - negiert
      [
      bool
      , id=
      1193
      ]
    - vorhanden
      [
      bool
      , id=
      1194
      ]
    - bei Belastung
      [
      bool
      , id=
      983
      ]
  - echokardiographischer Befund
    [
    attribute
    , id=
    907
    ]
    - normal
      [
      bool
      , id=
      908
      ]
    - im übrigen normal
      [
      bool
      , id=
      1437
      ]
  - Untersuchungen
    [
    structure
    , id=
    1564
    ]
    - CT
      [
      attribute
      , id=
      1563
      , templates="
      Default Untersuchung
      "
      ]
    - TEE
      [
      attribute
      , id=
      1528
      , templates="
      Default Untersuchung
      "
      ]
      - vorhanden
        [
        bool
        , id=
        1529
        ]
    - cardio CT
      [
      attribute
      , id=
      1565
      , templates="
      Default Untersuchung
      "
      ]
- Voelker (min)
  [
  structure
  , id=
  1530
  ]
  - Allgemeine Angaben
    [
    structure
    , id=
    1531
    ]
    - Schallbarkeit
      [
      object
      , id=
      663
      ]
      - Parasternale Schallbarkeit
        [
        attribute
        , id=
        664
        ]
        - gut
          [
          bool
          , id=
          667
          ]
        - schlecht
          [
          bool
          , id=
          768
          ]
        - zufriedenstellend
          [
          bool
          , id=
          769
          ]
        - Nicht durchgeführt
          [
          bool
          , id=
          1262
          ]
      - apikale Schallbarkeit
        [
        attribute
        , id=
        665
        ]
        - gut
          [
          bool
          , id=
          668
          ]
        - schlecht
          [
          bool
          , id=
          770
          ]
        - zufriedenstellend
          [
          bool
          , id=
          771
          ]
        - nicht durchgeführt
          [
          bool
          , id=
          1261
          ]
      - subcostale Schallbarkeit
        [
        attribute
        , id=
        666
        ]
        - gut
          [
          bool
          , id=
          669
          ]
        - schlecht
          [
          bool
          , id=
          772
          ]
        - zufriedenstellend
          [
          bool
          , id=
          773
          ]
        - nicht durchgeführt
          [
          bool
          , id=
          1263
          ]
      - Zustand
        [
        object\_attribute
        , id=
        1308
        ]
        - schlecht
          [
          bool
          , id=
          1309
          ]
  - Aortenklappe
    [
    object
    , id=
    1000
    ]
    - Flussgeschwindigkeit
      [
      object\_attribute
      , id=
      1237
      ]
      - negiert
        [
        bool
        , id=
        1542
        ]
      - vorhanden
        [
        bool
        , id=
        1543
        ]
      - normal
        [
        bool
        , id=
        1574
        ]
    - Zustand
      [
      object\_attribute
      , id=
      1239
      ]
      - unauffällig
        [
        bool
        , id=
        1001
        ]
      - nicht beurteilbar
        [
        bool
        , id=
        1164
        ]
      - Bikuspide Anlage
        [
        bool
        , id=
        1002
        ]
      - kalkdicht
        [
        bool
        , id=
        1240
        ]
      - hyperdens
        [
        bool
        , id=
        1241
        ]
      - verdickt
        [
        bool
        , id=
        1497
        ]
    - Stenose
      [
      object\_attribute\_plus
      , id=
      1249
      , templates="
      Schweregrade
      "
      ]
      - negiert
        [
        bool
        , id=
        1250
        ]
      - verkalkt
        [
        bool
        , id=
        1353
        ]
      - vorhanden
        [
        bool
        , id=
        1360
        ]
    - Trikuspide Anlage
      [
      attribute\_plus
      , id=
      1311
      ]
      - negiert
        [
        bool
        , id=
        1312
        ]
      - vorhanden
        [
        bool
        , id=
        1313
        ]
    - Aortenklappenprotheseninsuffizienz
      [
      attribute\_plus
      , id=
      1319
      , templates="
      Insuffizienz
      "
      ]
      - negiert
        [
        bool
        , id=
        1320
        ]
      - vorhanden
        [
        bool
        , id=
        1321
        ]
    - Mobilität
      [
      object\_attribute
      , id=
      1341
      , templates="
      Mobilitaet
      "
      ]
    - Aortenklappensklerose
      [
      attribute\_plus
      , id=
      1357
      ]
      - negiert
        [
        bool
        , id=
        1358
        ]
      - vorhanden
        [
        bool
        , id=
        1359
        ]
    - Insuffizienz
      [
      object\_attribute\_plus
      , id=
      870
      , templates="
      Insuffizienz
      "
      ]
      - negiert
        [
        bool
        , id=
        1177
        ]
      - vorhanden
        [
        bool
        , id=
        1178
        ]
    - AÖF
      [
      attribute
      , id=
      1344
      , templates="
      Default Messung\_qcm
      "
      ]
      Aortenklappenöffnungsfläche
      - eingeschränkt
        [
        bool
        , id=
        1514
        ]
      - normal
        [
        bool
        , id=
        1512
        ]
      - planimetrisch
        [
        bool
        , id=
        1513
        ]
    - AV-Pmax
      [
      attribute
      , id=
      630
      , templates="
      Messung\_mmHg
      "
      ]
      maximaler Druckgradient
    - Aortenklappenfibrose
      [
      attribute\_plus
      , id=
      987
      ]
      - negiert
        [
        bool
        , id=
        1189
        ]
      - vorhanden
        [
        bool
        , id=
        1190
        ]
      - leicht
        [
        bool
        , id=
        988
        ]
      - nicht beurteilbar
        [
        bool
        , id=
        1162
        ]
    - Öffnungsbewegung
      [
      object\_attribute
      , id=
      1332
      , templates="
      Mobilitaet
      "
      ]
    - dPmax
      [
      attribute
      , id=
      684
      ]
      maximaler Druckgradient
      - Messwert mmHg
        [
        numeric\_eq
        , id=
        685
        ]
    - AV-Vmax
      [
      attribute
      , id=
      628
      , templates="
      Messung\_m\_per\_s
      "
      ]
      maximale systolische Geschwindigkeit
    - Aortentaschenfibrose
      [
      attribute\_plus
      , id=
      764
      ]
      - negiert
        [
        bool
        , id=
        1173
        ]
      - vorhanden
        [
        bool
        , id=
        1174
        ]
      - leicht
        [
        bool
        , id=
        981
        ]
      - nicht beurteilbar
        [
        bool
        , id=
        1150
        ]
    - Klappenvegetationen
      [
      attribute\_plus
      , id=
      1452
      ]
      - negiert
        [
        bool
        , id=
        1453
        ]
      - vorhanden
        [
        bool
        , id=
        1454
        ]
  - Trikuspidalklappe
    [
    object
    , id=
    1008
    ]
    - Zustand
      [
      object\_attribute
      , id=
      1302
      ]
      - nicht beurteilbar
        [
        bool
        , id=
        1010
        ]
      - unauffällig
        [
        bool
        , id=
        1009
        ]
    - Öffnungsfläche
      [
      object\_attribute
      , id=
      1523
      ]
      - normal
        [
        bool
        , id=
        1524
        ]
      - planimetrisch
        [
        bool
        , id=
        1525
        ]
      - eingeschränkt
        [
        bool
        , id=
        1526
        ]
    - Insuffizienz
      [
      object\_attribute\_plus
      , id=
      742
      , templates="
      Insuffizienz
      "
      ]
      - negiert
        [
        bool
        , id=
        1169
        ]
      - vorhanden
        [
        bool
        , id=
        1170
        ]
      - holosystolisch
        [
        bool
        , id=
        800
        ]
    - sPAP
      [
      attribute
      , id=
      640
      , templates="
      Messung\_mmHg
      "
      ]
      systolischer pulmonal arterieller Druck
      - normal
        [
        bool
        , id=
        721
        ]
      - grenzwertig
        [
        bool
        , id=
        899
        ]
      - erhöht
        [
        bool
        , id=
        909
        ]
      - nicht beurteilbar
        [
        bool
        , id=
        1166
        ]
    - Mobilität
      [
      object\_attribute
      , id=
      1560
      , templates="
      Default Mobilitaet
      "
      ]
  - Mitralklappe
    [
    object
    , id=
    1006
    ]
    - Flussgeschwindigkeit
      [
      object\_attribute
      , id=
      698
      ]
      - normal
        [
        bool
        , id=
        701
        ]
    - Zustand
      [
      object\_attribute
      , id=
      1225
      ]
      - nicht beurteilbar
        [
        bool
        , id=
        1142
        ]
      - Reflux
        [
        bool
        , id=
        700
        ]
      - unauffällig
        [
        bool
        , id=
        689
        ]
    - Segel
      [
      attribute
      , id=
      696
      ]
      - hyperdens
        [
        bool
        , id=
        697
        ]
      - verdickt
        [
        bool
        , id=
        1401
        ]
    - Stenose
      [
      object\_attribute\_plus
      , id=
      1283
      , templates="
      Insuffizienz
      "
      ]
      - negiert
        [
        bool
        , id=
        1284
        ]
      - vorhanden
        [
        bool
        , id=
        1285
        ]
    - Öffnungsfläche
      [
      object\_attribute
      , id=
      1515
      ]
      - normal
        [
        bool
        , id=
        1516
        ]
      - planimetrisch
        [
        bool
        , id=
        1517
        ]
      - eingeschränkt
        [
        bool
        , id=
        1518
        ]
    - Insuffizienz
      [
      object\_attribute\_plus
      , id=
      699
      , templates="
      Default Insuffizienz
      "
      ]
      - negiert
        [
        bool
        , id=
        1544
        ]
      - vorhanden
        [
        bool
        , id=
        1545
        ]
      - nicht beurteilbar
        [
        bool
        , id=
        1143
        ]
      - holosystolisch
        [
        bool
        , id=
        787
        ]
    - MÖF
      [
      attribute
      , id=
      1370
      , templates="
      Messung\_qcm
      "
      ]
      Mitralöffnungsfläche
    - Mitralringverkalkung
      [
      attribute\_plus
      , id=
      922
      , templates="
      Schweregrade
      "
      ]
      - posterior
        [
        bool
        , id=
        926
        ]
      - anterior
        [
        bool
        , id=
        927
        ]
      - negiert
        [
        bool
        , id=
        1230
        ]
      - vorhanden
        [
        bool
        , id=
        1231
        ]
    - MI-Jet
      [
      object\_attribute
      , id=
      788
      ]
      - posterior
        [
        bool
        , id=
        789
        ]
      - anterior
        [
        bool
        , id=
        790
        ]
      - zentral
        [
        bool
        , id=
        791
        ]
      - ohne Eigenschaft
        [
        bool
        , id=
        792
        ]
      - bis PV
        [
        bool
        , id=
        1234
        ]
    - Mobilität
      [
      object\_attribute
      , id=
      1561
      , templates="
      Default Mobilitaet
      "
      ]
  - Perikard
    [
    structure
    , id=
    1532
    ]
    - Perikarderguss
      [
      attribute\_plus
      , id=
      744
      , templates="
      Schweregrade
      "
      ]
      - negiert
        [
        bool
        , id=
        1171
        ]
      - vorhanden
        [
        bool
        , id=
        1172
        ]
      - nicht beurteilbar
        [
        bool
        , id=
        1149
        ]
  - linker Ventrikel (LV)
    [
    object
    , id=
    1533
    ]
    - LV-Zustand
      [
      object\_attribute
      , id=
      723
      , templates="
      Ventrikel
      "
      ]
    - LV-Durchmesser enddiastol. (LVDd)
      [
      attribute
      , id=
      618
      , templates="
      Messung\_mm
      "
      ]
      linksventrikulärer Durchmesser enddiastolisch
    - LV-Durchmesser endsystol. (LVDs)
      [
      attribute
      , id=
      620
      , templates="
      Messung\_mm
      "
      ]
      linksventrikulärer Durchmesser endsystolisch
    - Septum-Durchm. diastol. (IVSd)
      [
      attribute
      , id=
      622
      , templates="
      Messung\_mm
      "
      ]
      linksventrikuläre diastolische Septumdicke
    - Wanddicke (LVPWd)
      [
      object\_attribute
      , id=
      623
      , templates="
      Messung\_mm Wanddicke
      "
      ]
      linksventrikuläre diastolische Hinterwanddicke
    - LVEF
      [
      attribute
      , id=
      624
      , templates="
      Messwert\_Prozent
      "
      ]
      linksventrikuläre Ejektionsfraktion
      - visuell geschätzt
        [
        bool
        , id=
        648
        ]
      - Simpson biplan
        [
        bool
        , id=
        1025
        ]
    - Akinesie
      [
      attribute\_plus
      , id=
      967
      ]
      - negiert
        [
        bool
        , id=
        1376
        ]
      - vorhanden
        [
        bool
        , id=
        1377
        ]
    - Dyskinesie
      [
      attribute\_plus
      , id=
      1384
      ]
      - negiert
        [
        bool
        , id=
        1385
        ]
      - vorhanden
        [
        bool
        , id=
        1386
        ]
    - Hypokinesie
      [
      attribute\_plus
      , id=
      857
      ]
      - negiert
        [
        bool
        , id=
        1378
        ]
      - vorhanden
        [
        bool
        , id=
        1379
        ]
    - Linksventrikuläre Funktion
      [
      attribute
      , id=
      732
      , templates="
      Systolische\_Funktion
      "
      ]
      - kompensiert
        [
        bool
        , id=
        833
        ]
    - Diastolische Funktion
      [
      attribute
      , id=
      733
      ]
      - linksventrikulär
        [
        bool
        , id=
        736
        ]
      - rechtsventrikulär
        [
        bool
        , id=
        737
        ]
      - normal
        [
        bool
        , id=
        739
        ]
      - leichtgradig eingeschränkt
        [
        bool
        , id=
        836
        ]
      - nicht beurteilbar
        [
        bool
        , id=
        837
        ]
      - mittelgradig eingeschränkt
        [
        bool
        , id=
        838
        ]
      - hochgradig eingeschränkt
        [
        bool
        , id=
        839
        ]
      - Pseudonormales Füllungsmuster
        [
        attribute\_plus
        , id=
        935
        ]
        - negiert
          [
          bool
          , id=
          1351
          ]
        - vorhanden
          [
          bool
          , id=
          1352
          ]
      - Restriktives Füllungsmuster
        [
        attribute\_plus
        , id=
        936
        ]
        - negiert
          [
          bool
          , id=
          1349
          ]
        - vorhanden
          [
          bool
          , id=
          1350
          ]
      - mittel- bis hochgradig eingeschränkt
        [
        bool
        , id=
        1113
        ]
      - leicht- bis mittelgradige eingeschränkt
        [
        bool
        , id=
        1114
        ]
      - Relaxationsstörung
        [
        attribute\_plus
        , id=
        740
        ]
        - negiert
          [
          bool
          , id=
          1195
          ]
        - vorhanden
          [
          bool
          , id=
          1196
          ]
  - linker Vorhof (LA)
    [
    object
    , id=
    1534
    ]
    - LV Zustand
      [
      object\_attribute
      , id=
      726
      , templates="
      Vorhof
      "
      ]
    - LADs
      [
      attribute
      , id=
      626
      , templates="
      Messung\_mm
      "
      ]
      Left Atrium Diameter, systolic
    - LADsI
      [
      attribute
      , id=
      1394
      , templates="
      Messung\_m\_pro\_mm2
      "
      ]
      left atrial diameter septal-lateral
  - Aorta
    [
    object
    , id=
    1205
    ]
    - Wanddurchmesser
      [
      attribute
      , id=
      1219
      ]
      - Wert kleiner (mm)
        [
        numeric\_lt
        , id=
        1220
        ]
    - Aortenektasie
      [
      attribute\_plus
      , id=
      1354
      ]
      - negiert
        [
        bool
        , id=
        1355
        ]
      - vorhanden
        [
        bool
        , id=
        1356
        ]
    - Aortenbulbusektasie
      [
      attribute\_plus
      , id=
      1361
      ]
      - negiert
        [
        bool
        , id=
        1362
        ]
      - vorhanden
        [
        bool
        , id=
        1363
        ]
    - Regurgitationsvolumen
      [
      attribute
      , id=
      1371
      , templates="
      Messung\_ml
      "
      ]
    - Öffnungsfläche
      [
      object\_attribute
      , id=
      1492
      , templates="
      Schweregrade
      "
      ]
      - normal
        [
        bool
        , id=
        1493
        ]
      - planimetrisch
        [
        bool
        , id=
        1494
        ]
      - eingeschränkt
        [
        bool
        , id=
        1495
        ]
    - Aortensklerose
      [
      attribute\_plus
      , id=
      1508
      ]
      - negiert
        [
        bool
        , id=
        1509
        ]
      - vorhanden
        [
        bool
        , id=
        1510
        ]
    - Ao-root
      [
      attribute
      , id=
      627
      , templates="
      Messung\_mm
      "
      ]
      - ektatisch
        [
        bool
        , id=
        775
        ]
      - normal weit
        [
        bool
        , id=
        1020
        ]
    - Zustand
      [
      object\_attribute
      , id=
      1224
      ]
      - hyperdens
        [
        bool
        , id=
        1218
        ]
      - ohne pathologischen Befund
        [
        bool
        , id=
        1275
        ]
  - rechter Ventrikel (RV)
    [
    object
    , id=
    1535
    ]
    - rechter Ventrikel
      [
      object\_attribute
      , id=
      746
      , templates="
      Ventrikel
      "
      ]
    - Wanddicke
      [
      object\_attribute
      , id=
      1580
      , templates="
      Default Wanddicke
      "
      ]
- Voelker (max)
  [
  structure
  , id=
  1536
  ]
  - Linker Ventrikel
    [
    structure
    , id=
    1537
    ]
    - LVMI
      [
      attribute
      , id=
      1416
      ]
      linksventrikulärer Muskelmasseindex
      - Messung in g/qm
        [
        bool
        , id=
        1575
        ]
    - Muskelmasseindex
      [
      attribute
      , id=
      945
      ]
      - linksventrikulär
        [
        bool
        , id=
        946
        ]
      - normal
        [
        bool
        , id=
        947
        ]
      - nicht beurteilbar
        [
        bool
        , id=
        1161
        ]
      - rechtsventrikulär
        [
        bool
        , id=
        1314
        ]
  - Aortenklappe
    [
    structure
    , id=
    1538
    ]
    - AV-Pmean
      [
      attribute
      , id=
      631
      , templates="
      Messung\_mmHg
      "
      ]
      mittlerer transvalvuläre Druckgradient
    - AV-Vmean
      [
      attribute
      , id=
      629
      , templates="
      Messung\_m\_per\_s
      "
      ]
      mittlere Systolische Geschwindigkeit

### Templates

- Messung [mm]
  [
  template
  , id=
  -1
  ]
  - Wert (mm)
    [
    numeric\_eq
    , id=
    1267
    ]
- Insuffizienz
  [
  template
  , id=
  -1
  ]
  - physiologisch
    [
    bool
    , id=
    1365
    ]
- Messung [ms]
  [
  template
  , id=
  -1
  ]
  - Messung msec
    [
    numeric\_eq
    , id=
    978
    ]
- Messung [mmHg]
  [
  template
  , id=
  -1
  ]
  - Messwert mmHg
    [
    numeric\_eq
    , id=
    651
    ]
  - Messwertbereich [mmHg]
    [
    numeric\_from\_to
    , id=
    1347
    ]
  - Verhältnis [mmHg]
    [
    numeric\_eq
    , id=
    1371
    ]
- Messung [Prozent]
  [
  template
  , id=
  -1
  ]
  - Messwert % gleich
    [
    numeric\_eq
    , id=
    643
    ]
  - Messwert % größer
    [
    numeric\_gt
    , id=
    644
    ]
- Vorhof
  [
  template
  , id=
  -1
  ]
- Ventrikel
  [
  template
  , id=
  -1
  ]
- Höhle
  [
  template
  , id=
  -1
  ]
  - hochgradig dilatiert
    [
    bool
    , id=
    1285
    ]
  - leicht- bis mittelgradig dilatiert
    [
    bool
    , id=
    1286
    ]
  - leichtgradig dilatiert
    [
    bool
    , id=
    1287
    ]
  - Messung mm
    [
    numeric\_eq
    , id=
    1288
    ]
  - mittel- bis hochgradig dilatiert
    [
    bool
    , id=
    1289
    ]
  - mittelgradig dilatiert
    [
    bool
    , id=
    1290
    ]
  - nicht beurteilbar
    [
    bool
    , id=
    1291
    ]
  - normal groß
    [
    bool
    , id=
    1292
    ]
  - o.p.B.
    [
    bool
    , id=
    1293
    ]
- Systolische Funktion
  [
  template
  , id=
  -1
  ]
  - hochgradig eingeschränkt
    [
    bool
    , id=
    1294
    ]
  - leicht- bis mittelgradige eingeschränkt
    [
    bool
    , id=
    1295
    ]
  - leichtgradig eingeschränkt
    [
    bool
    , id=
    1296
    ]
  - mittel- bis hochgradig eingeschränkt
    [
    bool
    , id=
    1297
    ]
  - mittelgradig eingeschränkt
    [
    bool
    , id=
    1298
    ]
  - nicht beurteilbar
    [
    bool
    , id=
    1299
    ]
  - niedrig-normal
    [
    bool
    , id=
    1300
    ]
  - normal
    [
    bool
    , id=
    1301
    ]
- Schweregrade
  [
  template
  , id=
  -1
  ]
  - hochgradig
    [
    bool
    , id=
    1302
    ]
  - leicht- bis mittelgradig
    [
    bool
    , id=
    1303
    ]
  - leichtgradig
    [
    bool
    , id=
    1304
    ]
  - mittel- bis hochgradig
    [
    bool
    , id=
    1305
    ]
  - mittelgradig
    [
    bool
    , id=
    1306
    ]
  - nicht beurteilbar
    [
    bool
    , id=
    1307
    ]
  - nicht signifikant
    [
    bool
    , id=
    1367
    ]
- Segmente
  [
  template
  , id=
  -1
  ]
  - anterior
    [
    bool
    , id=
    1308
    ]
  - anterior basal
    [
    bool
    , id=
    1309
    ]
  - anterior medial
    [
    bool
    , id=
    1310
    ]
  - anterolateral
    [
    bool
    , id=
    1311
    ]
  - anterolateral apikal
    [
    bool
    , id=
    1312
    ]
  - anterolateral basal
    [
    bool
    , id=
    1313
    ]
  - anterolateral medial
    [
    bool
    , id=
    1314
    ]
  - anteroseptal
    [
    bool
    , id=
    1315
    ]
  - anteroseptal apikal
    [
    bool
    , id=
    1316
    ]
  - anteroseptal basal
    [
    bool
    , id=
    1317
    ]
  - anteroseptal medial
    [
    bool
    , id=
    1318
    ]
  - apikal
    [
    bool
    , id=
    1319
    ]
  - apikal anterior
    [
    bool
    , id=
    1320
    ]
  - apikal inferior
    [
    bool
    , id=
    1321
    ]
  - apikal lateral
    [
    bool
    , id=
    1322
    ]
  - apikal septal
    [
    bool
    , id=
    1323
    ]
  - global
    [
    bool
    , id=
    1324
    ]
  - inferior
    [
    bool
    , id=
    1325
    ]
  - inferior basal
    [
    bool
    , id=
    1326
    ]
  - inferior medial
    [
    bool
    , id=
    1327
    ]
  - inferolateral
    [
    bool
    , id=
    1328
    ]
  - inferolateral apikal
    [
    bool
    , id=
    1329
    ]
  - inferolateral basal
    [
    bool
    , id=
    1330
    ]
  - inferolateral medial
    [
    bool
    , id=
    1331
    ]
  - inferoseptal
    [
    bool
    , id=
    1332
    ]
  - inferoseptal apikal
    [
    bool
    , id=
    1333
    ]
  - inferoseptal basal
    [
    bool
    , id=
    1334
    ]
  - inferoseptal medial
    [
    bool
    , id=
    1335
    ]
  - medial
    [
    bool
    , id=
    1337
    ]
  - posterior
    [
    bool
    , id=
    1338
    ]
  - apikal posterior
    [
    bool
    , id=
    1340
    ]
  - posterior basal
    [
    bool
    , id=
    1351
    ]
  - septal basal
    [
    bool
    , id=
    1352
    ]
  - septal medial
    [
    bool
    , id=
    1354
    ]
  - lateral medial
    [
    bool
    , id=
    1355
    ]
  - lateral basal
    [
    bool
    , id=
    1356
    ]
  - lateral
    [
    bool
    , id=
    1358
    ]
  - septal
    [
    bool
    , id=
    1359
    ]
  - bis Spitze
    [
    bool
    , id=
    1360
    ]
- Messung (semantic class)
  [
  template
  , id=
  -1
  ]
- Label\_Anatomie
  [
  template
  , id=
  -1
  ]
- Label\_Symptom
  [
  template
  , id=
  -1
  ]
- Label\_Diagnose
  [
  template
  , id=
  -1
  ]
- Messung [mm²]
  [
  template
  , id=
  -1
  ]
  - Messwert [mm²]
    [
    numeric\_eq
    , id=
    1341
    ]
- Messung [m²]
  [
  template
  , id=
  -1
  ]
  - Messwert [m²]
    [
    numeric\_eq
    , id=
    1342
    ]
- Messung [m/s]
  [
  template
  , id=
  -1
  ]
  - Messwert m/s
    [
    numeric\_eq
    , id=
    1343
    ]
- Label\_Messung
  [
  template
  , id=
  -1
  ]
- Messung [cm]
  [
  template
  , id=
  -1
  ]
  - Messwert [cm]
    [
    numeric\_eq
    , id=
    1344
    ]
- Messung [ml/qm]
  [
  template
  , id=
  -1
  ]
  - Messwert [ml/qm]
    [
    numeric\_eq
    , id=
    1346
    ]
- Messung [cm/s]
  [
  template
  , id=
  -1
  ]
  - Messwert [cm/s]
    [
    numeric\_eq
    , id=
    1348
    ]
- Messung [cm²]
  [
  template
  , id=
  -1
  ]
  - Messwert [cm²]
    [
    numeric\_eq
    , id=
    1349
    ]
- Messung [ml]
  [
  template
  , id=
  -1
  ]
  - Wert (ml)
    [
    numeric\_eq
    , id=
    1350
    ]
- Messung [mm/m²]
  [
  template
  , id=
  -1
  ]
  - Wert [mm/m2]
    [
    numeric\_eq
    , id=
    1361
    ]
- Mobilität
  [
  template
  , id=
  -1
  ]
  - normal
    [
    bool
    , id=
    1362
    ]
  - eingeschränkt
    [
    bool
    , id=
    1363
    ]
- Untersuchung
  [
  template
  , id=
  -1
  ]
  - empfohlen
    [
    bool
    , id=
    1366
    ]
- Wanddicke
  [
  template
  , id=
  -1
  ]
  - normal
    [
    bool
    , id=
    1368
    ]
  - gering verdickt
    [
    bool
    , id=
    1369
    ]
  - verdickt
    [
    bool
    , id=
    1370
    ]
